# Sex-Specific Response to Caloric Restriction After Reproductive Investment in *Microcebus murinus*: An Integrative Approach

**DOI:** 10.3389/fphys.2020.00506

**Published:** 2020-06-16

**Authors:** Aude Noiret, Laura Puch, Coralie Riffaud, David Costantini, Jean-Francois Riou, Fabienne Aujard, Jeremy Terrien

**Affiliations:** ^1^Unité Mécanismes Adaptatifs et Evolution (MECADEV), Muséum National d'Histoire Naturelle, CNRS UMR 7179, Brunoy, France; ^2^Unité Physiologie Moléculaire et Adaptation (PhyMA), Muséum National d'Histoire Naturelle, CNRS UMR 7221, Paris, France; ^3^Unité Structure et Instabilité des Génomes (STRING), Muséum National d'Histoire Naturelle, CNRS UMR 7196, INSERM U1154, Paris, France

**Keywords:** caloric restriction, sex, season, reproductive investment, oxidative stress, non-human primate

## Abstract

In seasonal environments, males and females usually maintain high metabolic activity during the whole summer season, exhausting their energy reserves. In the global warming context, unpredictability of food availability during summer could dramatically challenge the energy budget of individuals. Therefore, one can predict that resilience to environmental stress would be dramatically endangered during summer. Here, we hypothesized that females could have greater capacity to survive harsh conditions than males, considering the temporal shift in their respective reproductive energy investment, which can challenge them differently, as well as enhanced flexibility in females' physiological regulation. We tackled this question on the gray mouse lemur (*Microcebus murinus*), focusing on the late summer period, after the reproductive effort. We monitored six males and six females before and after a 2-weeks 60% caloric restriction (CR), measuring different physiological and cellular parameters in an integrative and comparative multiscale approach. Before CR, females were heavier than males and mostly characterized by high levels of energy expenditure, a more energetic mitochondrial profile and a downregulation of blood antioxidants. We observed a similar energy balance between sexes due to CR, with a decrease in metabolic activity over time only in males. Oxidative damage to DNA was also reduced by different pathways between sexes, which may reflect variability in their physiological status and life-history traits at the end of summer. Finally, females' mitochondria seemed to exhibit greater flexibility and greater metabolic potential than males in response to CR. Our results showed strong differences between males and females in response to food shortage during late summer, underlining the necessity to consider sex as a factor for population dynamics in climate change models.

## Introduction

In highly seasonal environments, reproduction has evolved to be restrained to the most energetically favorable period to optimize young growth (Fournier et al., 1999), which is a fragile and high-stake phase though crucial to ensure population renewal (Wells, 2000). But with more frequent climatic disturbances related to global warming, there is no longer certainty that summer will support such energetic demands (Canale and Henry, 2010). This season might therefore become a risky period, where animals, exhausted after their reproductive effort, would be more vulnerable to environmental change and become unable to compensate unexpected altered food availability. Additionally, individuals forage to fill up their energy storages in late summer to face winter's own harsh conditions, such as dryness and cold temperatures (Dammhahn and Kappeler, 2008), but could face instead their physiological limit to prolonged environmental stress.

In most mammal species, males and females are invested in specific physiological tasks regarding reproduction, as males produce high quantities of spermatozoa, whereas females ovulate, complete gestation, and lactation. With a large panel of peculiarities in the animal kingdom, from mating strategies to young care sharing or even hermaphroditism, sex-specific behavior and physiology can describe little to huge dimorphism amongst species (Trivers, 1972; Zeveloff and Boyce, 1980). Additionally, seasonality could have contributed to establish a phenological shift in the energetic investment for reproduction between males and females. Indeed, males use their fat stocks for spermatogenesis, a long-standing and costly process (Dewsbury, 1982; Thomsen et al., 2006), obligating males to trigger the production of sperm by anticipation of the mating season, when food availability is still low. In contrast, females mostly invest afterwards, when the environment is energetically favorable (Trivers, 1972; Zeveloff and Boyce, 1980). The ecological “niche” in which animals enter an active metabolic status can thus drastically differ between sexes (Shine, 1989) and could expose males and females to unequal challenges. Moreover, there is growing evidence of sex-specific physiological responses to poor food conditions in various species, which are generally hinged on the reproduction event. Heterothermic females can express hypometabolism in anticipation of the mating season (Humphries et al., 2003; Jonasson and Willis, 2011; CZenze et al., 2017), during gestation and lactation (Lennox and Goodship, 2008; McAllan and Geiser, 2014), or present general self-preserving abilities (Rodriguez-Cuenca et al., 2002; Valle et al., 2007). In a context of more frequent unpredictable climatic disturbances, we ask the question of a sex-imbalance in the trade-off between reproduction and survival and consider the hypothesis of a greater exhaustion in males than in females at the end of summer.

The gray mouse lemur (*Microcebus murinus)* can provide experimental evidence for a sex-biased response to food shortage in summer, as it is a highly seasonal Malagasy primate, able to enter deep winter torpors (Genin and Perret, 2003; Giroud et al., 2008). Although this species is considered monomorphic, males and females express strong differences in their seasonal weight curves, as males begin to lose body mass in the middle of winter concomitantly to testicular growth (Perret and Aujard, 2001; Terrien et al., 2017) but females keep their fat storage until summer when mating takes place. Male mouse lemurs show unusual extreme large testes relative to their body size and display characteristics associated with sperm competition (high spermatogenic efficacy and motility and low percentages of defect) (Harcourt et al., 1981; Aslam et al., 2002). These features are likely to demand strong energy expenditure (Dewsbury, 1982; Wedell et al., 2002; Thomsen et al., 2006), especially since males continue to maintain sexual activity late during the 6 months long-day period (Perret and Aujard, 2001).

A previous food shortage experiment showed that male mouse lemurs can maintain their body mass under a 40% caloric restriction during short day exposure, by increasing their torpor depth and duration, while the same treatment during long days induced mass loss (Giroud et al., 2008). In another study, a 40% food restriction during summer altered learning abilities of males, which surprisingly showed increased resting metabolic rate and no change in activity patterns (Villain et al., 2016). No experimental study has yet formally compared physiological responses to food shortage between sexes, although females were already described to use deeper and longer torpor than males to face food rarefaction at the onset of winter season in field conditions (Vuarin et al., 2015). Females are receptive to copulation for a short period of a few days during the estrus at the vernal equinox in natural conditions or after the photoperiodic change to long-days in breeding colonies (Perret and Aujard, 2001). Moreover, they can maintain their body mass during gestation with small variation of food intake in captive conditions (Wrogemann et al., 2001; Canale et al., 2012a), and are also able to use torpor during gestation but lose weight when lactation occurs regardless of the feeding regimen (Canale et al., 2012b). The reproductive resilience of mouse lemur females to food shortage, i.e., their ability to restore reproductive success after an environmental pressure, seems to remain during the flexible period of lactation, which only impacts litter growth in restricted individuals, while reproductive success stays unchanged compared to *ad-libitum* mothers (Canale et al., 2012a). No field data comparing sexes allows us however to infer about a possible energy exhaustion of males at the end of summer in natural conditions. But we do observe a sex-imbalance in body mass in the breeding colony of Brunoy at this particular period, though animals remain under normal feeding regimen (94.5 ± 2.24 g vs. 86.7 ± 1.99 g for females and males, respectively; *p* < 0.001; unpublished data). This observation strengthens the hypothesis of a rougher summer for males (although animals are not energetically exhausted), even in the absence of environmental perturbations.

For such heterogeneous physiological events occurring over the 6-months period of summer, males and females would likely express variable metabolic statuses and efficacy in their response to an unpredicted environmental stress. We thus tested the occurrence of sex-variability in energy management and its physiological impact on the well-suited model of *M. murinus*. We monitored six males and six females over a period of 2 weeks during which they experienced a 60% caloric restriction (CR) induced at the end of summer after all reproductive events. In an integrative and comparative (females vs. males) approach, we measured various energy-management parameters at different scales, from the whole organism (respiratory metabolism using indirect calorimetry, metabolic and sexual hormones) to the cellular level (mitochondrial respiration of cultured fibroblasts) and stress-related indicators (urinary and blood markers of oxidative damage and anti-oxidant activity) to better describe the physiological mechanisms modulated by CR from perception of the stress, to response and coping. We expected evidence of a higher impact of CR on males with different metabolic strategies of coping expressed between sexes.

## Materials and Methods

### Tested Animals and Ethical Concerns

Twelve gray mouse lemurs (*M. murinus*), 6 males and 6 females all aged from 1 to 5 years (mean age ± sd: 2 ± 1.1 years) and raised in good health in the breeding colony of Brunoy (MNHN, France, license approval n° E91-114-1), were included in the experiment. All females had just successfully raised a litter of three juveniles, which were weaned before starting the experiment, after 2 months of maternal care. All males had undergone at least one reproduction event, in the presence of one female and one or several males. These animals were thus tested at the late end of the summer-like reproductive season, after 5 months of long photoperiod exposure (14 h light/day), at the time when both sexes reached their minimal body weights (Perret and Aujard, 2001). Animals were kept in individual cages in semi-isolation from the others (visual, hearing and odorant interactions remaining possible between individuals) in climatic chambers for the duration of the experiment (1 month). Temperature and humidity were maintained constant (24–26°C and 55%, respectively). The lemurs were fed with a fresh mixture (egg, concentrate milk, cereals, spicy bread, cream cheese, and water), banana, and were provided with *ad libitum* water. All described experimental procedures were approved by the Animal Welfare board of the UMR 7179, the Cuvier Ethics Committee for the Care and Use of Experimental Animals of the Muséum National d'Histoire Naturelle and authorized by the Ministère de l'Enseignement Supérieur, de la Recherche et de l'Innovation (n°14075-2018031509218574) and complied with the European ethic regulations for the use of animals in biomedical research.

### Experimental Design

Individuals were designed to be their own control as their physiology in late summer is supposedly not varying as long as the environment remains stable (photoperiod, temperature, food availability) (Perret and Aujard, 2001). We performed an integrative description of physiological parameters, general and cellular scaled, to decipher the energy balance of the animals, their metabolic activity, and their oxidative regulation and damage. For this, animals were fed daily with a control ration (Control treatment “CTL”) of 22 g of mixture and 3 g of banana (24.48 kcal.day^−1^) for 1 week and measured during four consecutive days for indirect calorimetry (Oxymax, Columbus Instrument Inc., Columbus, Ohio, USA). At the end of this procedure, animals went through a series of non-invasive (urine) and invasive sampling (blood puncture via the saphenous vein, and abdominal skin biopsy after lidocaine injection to ensure local analgesia), always performed ~3 h prior to lights off. Urine samples were used to measure creatinine, cortisol, and 8-OHdG; blood samples were used to measure glycaemia, thyroxin hormone (T4) and antioxidant activity. Finally, skin biopsies were dedicated to develop primary fibroblastic cultures to assess mitochondrial activity. After 1 day of recovery, animals were fed daily with a 60% reduced ration compared to the CTL condition (Caloric Restriction “CR,” 9.79 kcal.day^−1^) for 2 weeks, inducing a sufficient caloric stress to mimic food shortage, but not as much to put animals at risk considering this time of the year where body weights can be low. They were monitored with weighing three times a week to follow their body mass (“BM” in g). At the end of the treatment, they underwent the same procedures as the control period (4-days indirect calorimetry, urine/blood/biopsy sampling). Animals returned under control diet for several days to allow a full recovery from the CR treatment and returned to the breeding colony in their original social groups.

### Indirect Calorimetry

Animals were put in monitored cages for 4 consecutive days to measure their consumption of O_2_ (“VO_2_” in ml.kg.h^−1^, see equation 1, Lighton, 2018) and production of CO_2_ (“VCO_2_” in ml.kg.hr^−1^, see equation 2, Lighton, 2018) in a continuous way using an automated calorimetric set-up (Oxymax, Colombus Instruments Inc., Columbus, Ohio, USA).

(1) $$VO_{2}=\frac{FR_{i}[\left(F_{i}O_{2}-F_{e^{\prime }}O_{2}\right)-F_{e^{\prime }}O_{2}\left(F_{e^{\prime }}CO_{2}-F_{i}CO_{2}\right)]}{\left(1-F_{e^{\prime }}O_{2}\right)\;m}$$

FR_i_ and FR_e_ are the incurrent and excurrent flow rates of all the separate gas species.

F_i_O_2_ (F_i_CO_2_) and F_e_O_2_ (F_e_CO_2_) are the fractional concentrations of the incurrent and excurrent O_2_ (or CO_2_).

F_e_'O_2_ (F_e_'CO_2_) are the fractional concentrations of excurrent O_2_ (CO_2_), scrubbed of water vapor. *m* is the body mass of the individual.

(2) $$VCO_{2}=\frac{FR_{i}[\left(F_{i}CO_{2}-F_{e^{\prime }}CO_{2}\right)-F_{e^{\prime }}CO_{2}\left(F_{e^{\prime }}O_{2}-F_{i}O_{2}\right)]}{\left(1-F_{e^{\prime }}CO_{2}\right)\;m}$$

Air was dried with magnesium perchlorate columns prior to analysis. As metabolic rate (“MR”) is acknowledged to be directly linked to oxygen consumption rate (Geiser et al., 2014) we used the VO_2_ parameter to describe variations in mouse lemurs' metabolism. Respiratory Exchange Ratio (“RER”) was calculated as the ratio of VCO_2_/VO_2_. This parameter allows to extrapolate the nature of energy substrates used for oxidative metabolism as described in Lusk's oxidation table (Lusk, 1924), RER varying between 1 (indicating a full carbohydrate energy substrate) and 0.7 (obtained when animals only depend on fat energy substrate). Energy expenditure (“EE,” kcal.h^−1^) was obtained as follows: EE = (3.815 + 1.232 × RER) × VO_2_ × BM, an equation that also derives from Lusk's table (Lusk, 1924). Analyses were based on mean parameters over day (period under artificial light) and night (period without artificial light). Maximum and minimum values of each parameter (“Max” and “Min”) were also obtained using the *changepoint* package *v2.2.2* (Killick and Eckley, 2014), to detect the significant maximum and minimum changes of the parameters transformed as time series. Time (“hMax” or “hMin” in hh:mm) at which Max and Min values were achieved was also analyzed and set as polar coordinates to be represented in 24 h circular diagrams.

### Urine Samples: Cortisol, 8-OHdG, 17-Beta-Estradiol and Testosterone

Cortisol (ng.ml^−1^; Cortisol Urine ELISA from LDN®, ref MS E-5100) and 8-hydroxy-2'-deoxyguanosine (“8-OHdG,” in ng.ml^−1^; OxiSelect™ Oxidative DNA Damage Elisa kit, Cell Biolabs Inc., ref STA-320) were measured in duplicates from urine samples as indicators of the organisms' response to environmental stress (Miller et al., 1991) and oxidative-stress related DNA damage (Loft et al., 1993), respectively. 17-beta-Estradiol (“17-beta-Estradiol” in pg.ml^−1^; 17beta-Estradiol, IBL, ref RE52041) and Testosterone (“Testosterone” in ng.ml^−1^; Testosterone ELISA kit from Abcam, ref ab108666) were also quantified in urine. Creatinine concentration (mg.ml^−1^) was used to normalize all urine measurements as an indicator of renal filtration activity (Microvue™ Creatinine Elisa kit, Quidel® Corporation, ref 8009). Results are thus expressed in ng.mg Creat.^−1^ or pg.mg Creat.^−1^.

### Blood Samples: Fasting Glycaemia, Thyroxin Hormone, and Antioxidant Machinery

Fasting glycaemia (mg.dl^−1^) was directly obtained at animals' bedside during blood sampling with a non-invasive glucometer (OneTouch® Vita glucometer, LifeScan, France). Measures were repeated twice when values were out of normal range. Thyroxin (“T4,” nmol.l^−1^) was assayed in plasma samples (T4 ELISA kit, LDN®, ref TF E-2400) and the 6-point standard curve was adapted from 0 to 100 nmol.l^−1^. Thiols (μmol.mgProtein^−1^, SHp Test, Diacron Labs srl, ref MC432) and Glutathione Peroxidase (“GPx,” U.mgProtein^−1^, RANSEL, RANDOX, with controls, ref RS 504) were assessed from haemolysates of the red blood cells pellets. These last two parameters were normalized with protein concentrations using Bradford method (for detailed method, see Costantini et al., 2017).

### Cellular Culture and Mitochondrial Analysis

Skin biopsies were washed in a series of PBS−70% alcohol—PBS baths and put in 50 μl 0.25% Trypsin solution (Gibco^TM^, ThermoFisher scientific) for a 1-h digestion. Each sample was then transferred in a 96-well plate to form a fibroblastic cell line, and maintained in DMEM/F-12 medium (DMEM/F-12, GlutaMAX^TM^ supplement, Gibco^TM^, ThermoFisher scientific) mixed with 10% fetal bovine serum, 1% Penicillin-Streptomycin (Penicillin-Streptomycin (5,000 U.ml^−1^), Gibco^TM^) and 0.1% Amphotericin B (Gibco™ Amphotericin B 250 μg.ml^−1^), in a 37°C incubator with 5% CO_2_. Cells underwent 4 passages by adding 0.25% trypsin for 10 min, from the 96-well plate to 24-, 12-, 6-well plates and finally a T75 flask. They were then counted on a Malassez cell and 100 000 cells of each cell line were put in a Seahorse XF24 Microplate (Agilent Technologies) in triplicates. The protocol followed the instructions of the XF Cell Mito Stress Test Kit and analysis was performed using the Wave Software (version 2.6.0.31, Agilent Technologies). The Oxidative Coupling Rate (“OxCR,” % of baseline OCR) was calculated as the difference between the mean of the 3 initial measures of oxygen consumption rate (“baseline OCR,” in pmole O_2_.min^−1^), and the minimum of the 3 measures following Oligomycin injection. Mitochondrial Reserve Capacity (“MtRC,” % baseline OCR) was obtained by substraction of the mean of the 3 initial measures to the maximum of the 3 measures following FCCP injection (“stressed OCR” in pmoleO_2_.min^−1^). These parameters were expressed as a percentage of the baseline OCR in order to reduce inter-well variability due to cell number bias. The Glycolytic Potential (“GlcP,” % of baseline ECAR) was extracted from ECAR measurements (in mpH.min^−1^), as the difference between the mean value obtained after FCCP injection (“stressed ECAR,” in mpH.min^−1^) and baseline ECAR (mean of the first three measures), expressed as a percentage of baseline ECAR. It represents the capacity of the cells to use glycolysis in order to produce ATP (Divakaruni et al., 2014). Finally, the Metabolic Potential is the gap between OCR / ECAR in basal conditions (“basal” cell phenotype) and OCR / ECAR after FCCP injection (“Stressed” cell phenotype) and informs on the general capacity of the cells to meet an induced energy demand either by glycolysis or oxidative respiration (referred as the metabolic potential of the cells).

After each assay, cells were trypsinized from each well and pellets were collected after centrifugation. Cells were lysed in PBS buffer containing Proteinase K (0.2 mg.ml^−1^), SDS (0.2%), EDTA (5 mM) for 3 h at 50°C. Total DNA was precipitated in 1.5 volume of cold ethanol with 0.2 M Sodium Acetate pH 5.2, and then centrifuged at 16,000 xg for 30 min at +4°C. After washing with 70% ethanol, pellet containing nuclear and mitochondrial DNA was resuspended in 10 mM Tris pH 8.0. DNA concentration was measured using fluorometry (Qubit™ 4 Fluorometer and Qubit™ dsDNA HS Assay Kit, Invitrogen™, ThermoFisher scientific). Mitochondrial DNA (mtDNA) and nuclear DNA (nuDNA) were quantified by qPCR using a 7300 Real-Time PCR System thermocycler (Applied Biosystems^TM^). Primers for mtDNA (12S) and nuDNA (36B4) were designed from the gray mouse lemur genome as follows and ordered to Eurogentec: 12S-R: 5′-TAGCAAGAGGGGGTGAGGTT-3′; 12S-F: 5′-CCACGACAGCCAAGATCCAA-3′; 36B4-R: 5′-CCCATTCTATCATCAATGGGTACAA-3′; 36B4-F: 5′-CAGCAAGTGGGAAGGTGTAATCA-3′. We used Power SYBR® Green MasterMix (Applied Biosystem^TM^, ThermoFisher scientific) as reaction media. The first thermic cycle was set to 95°C for 10 min, followed by 34 cycles of 95°C (30 s)−59°C (1 min)−72°C (1 min), then one final cycle of 95°C (1 min)-−55°C (30 s)−95°C (30 s). Amplification was measured at the last step of stage 2. Each sample was measured in two-time repeated triplicates and the mean value was used as data. The mtDNA/nuDNA ratio was used to account for intracellular contents in mitochondria.

### Statistical Analyses

Results shown are given as means ± standard deviation (s.d) in tables and as least square means (LSM) with 95% confidence intervals in Figures, except for T4 and GlcP whose modelization was too complex to be identifiable. All parameters were measured for the 12 animals at each regimen (*N* = 12), except for mitochondrial analysis due to contamination during cellular culture (*N* = 10 for CTL, *N* = 8 for CR regimen). No outlier was identified or removed from data sets after testing with Dixon's Q test. Statistical analysis was conducted by the use of R software v 3.5.1 (R Core Team, 2016), and tests were considered significant when *p*-values were below the significant level set at 0.05. We applied linear mixed models with random individual effect on parameters with normal distribution (see Table 2) to test the effect of sex, caloric restriction and their interaction. Body mass was included in models only when its effect on the explained variable was significant. Age had a significant effect on 8-OHdG only, and was thus included in the model. For non-Gaussian parameters, generalized mixed models with random effects were used when supported by the data sets (used family is written in Table 2). When not supported, non-parametric pairwise Wilcoxon test was used as a default analysis, as shown in Table 1, which gathered *post-hoc* analysis (pairwise non-parametric tests for caloric restriction effect, unpaired for sex or interaction effect). Pairwise correlations were performed using pooled data collected before and after CR for each parameter of each sex to better describe possible links between variables, and were represented into a network graphic using the “corrr” 3.5.3 package (Ruiz et al., 2019). Principal component analyses (either with females and males together, or by sex) were performed using the “FactominR” 1.34 package (Le et al., 2008), and missing values imputed with the “missMDA” 1.11 package (Josse and Husson, 2016).

**Table 1:** Mean and standard deviation of measured parameters for females (F) and males (M) in control treatment (ad libitum) and under 60% caloric restriction.

			**Control**		**Initial Sex effect**	**60% Caloric restriction**		**CR effect**		**Sex effect in the caloric restriction response**		
			***F***	***M***	***p-value***	***F***	***M***	***p-value F***	***p-value M***	***V°f (%)***	***V°m (%)***	***p-value***
Cortisol (ng.mgCreat.^−1^)			603.4 ± 317.8	403.2 ± 111.5	-	992.3 ± 604.3	596.4 ± 245.3	-	°	+87.3 ± 142.4	+43.5 ± 34.6	-
8-OHdG (ng.mgCreat. ^−1^)			387.4 ± 93.0	591.0 ± 262.4	°	214.1 ± 36.3	324.6 ± 110.8	*	*	−42.4 ± 14.9	−42.7 ± 12.8	-
Thiols (μmol.mgProt. ^−1^)			1.8 ± 0.5	2.2 ± 0.3	-	1.8 ± 0.6	1.4 ± 0.2	-	°	+4.9 ± 187.1	−29.8 ± 44.8	°
GPx (U.mgProt. ^−1^)			0.28 ± 0.10	0.25 ± 0.17	-	0.34 ± 0.06	0.14 ± 0.04	-	-	+28.85 ± 49.06	−41.08 ± 19.71	-
T4 (nmol.l^−1^)			30.6 ± 8.5	32.4 ± 3.2	-	25.8 ± 8.8	21.3 ± 10.0	-	*	−6.4 ± 46.6	−35.6 ± 27.4	-
17 beta-Estradiol (pg.mgCreat. ^−1^)			33558.8 ± 15819.7	23364.4 ± 8297.4	-	60086.8 ± 13681.9	40180.7 ± 14563.2	*	*	+119.9 ± 102.9	+74.4 ± 35.6	-
Testosterone (ng.mgCreat. ^−1^)			2.8 ± 1.9	64.1 ± 31.1	***	12.9 ± 16.7	94.4 ± 78.0	°	–	+667.1 ± 1023.8	+32.8 ± 65.7	*
Glycaemia (mg.dl^−1^)			95 ± 32	65 ± 9	*	84 ± 18	72 ± 11	-	-	−1 ± 51	+12 ± 26	°
Body mass (g)			99.7 ± 3.8	90.0 ± 6.6	*	83.0 ± 5.1	77.2 ± 5.4	*	*	−16.7 ± 4.3	−14.2 ± 4.3	°
Oxidative coupling rate (% of baseline OCR)			60.96 ± 6.6	72.59 ± 8.3	°	69.49 ± 2.3	67.54 ± 2.1	-	-	+9.77 ± 7.78	−2.97 ± 12.06	-
Mitochondrial reserve capacity (% of baseline OCR)			202.4 ± 45.9	152.0 ± 21.8	°	148.9 ± 33.0	134.8 ± 41.8	-	-	−23.63 ± 24.07	−19.7 ± 18.51	-
Glycolytic potential (% of baseline ECAR)			325.28 ± 27.37	363.92 ± 203.20	-	407.43 ± 128.65	439.77 ± 35.41	–	–	+35.34 ± 36.25	+44.70 ± 101.04	–
Mt/Nu DNA Ratio			1.78 ± 0.43	3.01 ± 0.44	*	2.56 ± 1.16	3.30 ± 1.43	-	-	+2.99 ± 25.79	+60.06 ± 30.17	°
RER	NIGHT	Max	1.01 ± 0.04	0.97 ± 0.07	-	0.91 ± 0.04	0.95 ± 0.02	*	-	−9.50 ± 4.49	–0.81 ± 6.78	*
		hMax	18:23 ± 1 h39 min	17:44 ± 1 h 34	-	17 h 30 ± 41 min	17 h 17 ± 41 min	-	-	−50 min ± 1 h34 min	−27 min ± 1 h52 min	-
		Mean	0.99 ± 0.077	0.96 ± 0.099	-	0.85 ± 0.076	0.90 ± 0.085	*	°	−13.0 ± 3.7	−4.9 ± 5.9	*
	DAY	Min	0.74 ± 0.05	0.73 ± 0.02	-	0.75 ± 0.02	0.71 ± 0.06	-	-	1.24 ± 7.07	−3.19 ± 7.01	-
		hMin	07:00 ± 03 h 45 min	10:43 ± 3 h 12 min	-	03:53 ± 4 h 35 min	4 h 58 ± 3 h 58 min	-	°	−3 h 08 min ± 5 h 08 min	−5 h45 ± 4 h 28 min	-
		Mean	0.77 ± 0.07	0.78 ± 0.07	-	0.76 ± 0.06	0.75 ± 0.07	-	*	−0.9 ± 5.2	−3.6 ± 1.8	-
VO_2_ (ml.kg^−1^.h^−1^)	NIGHT	Max	2,343 ± 124	2,287 ± 266	-	2,301 ± 194	2,228 ± 344	-	-	−1.7 ± 8.3	−1.8 ± 17.7	-
		hMax	19:36 ± 3 h 04 min	19:01 ± 3 h 07 min	-	18:55 ± 3 h 56 min	17:39 ± 2 h 13 min	-	°	−41 min ± 2 h 19 min	−1 h 22 min ± 2 h 15 min	-
		Mean	2,147 ± 403	2,192 ± 510	-	2,260 ± 432	1,882 ± 473	-	*	+7.7 ± 15.9	−10.2 ± 9.6	°
	DAY	Min	1,075 ± 135	1,183 ± 162	-	1,162 ± 198	758 ± 233	-	*	+8.2 ± 14.5	−35.6 ± 18.4	**
		hMin	07:59 ± 2 h 09 min	06:58 ± 4 h 32 min	-	05:50 ± 3 h 34	04:05 ± 1 h 08 min	-	-	−2 h 19 min ± 5 h 25 min	−2 h 53 min ± 4 h 42 min	-
		Mean	1226 ± 406	1296 ± 425	-	1328 ± 598	1001 ± 382	-	*	+9.6 ± 14.9	−9.4 ± 8.8	*
EE (kcal.h^−1^)	NIGHT	Max	1.18 ± 0.09	1.04 ± 0.17	-	1.06 ± 0.22	0.85 ± 0.12	-	*	−9.2 ± 32.6	−17.9 ± 7.8	-
		hMax	19:59 ± 3 h 01 min	20:27 ± 3 h 54	°	17:58 ± 1 h 38 min	18:08 ± 4 h	-	-	−2 h 02 min ± 1 h 58 min	−2 h 20 min ± 3 h 39 min	-
		Mean	1.06 ± 0.15	0.92 ± 0.16	°	0.93 ± 0.11	0.71 ± 0.06	°	*	−11.7 ± 10.8	−21.2 ± 11.6	-
	DAY	Min	0.50 ± 0.05	0.49 ± 0.07	-	0.45 ± 0.07	0.28 ± 0.09	-	*	−9.5 ± 16.1	−41.8 ± 18.4	*
		hMin	08:53 ± 28 min	08:15 ± 4 h 45 min	-	07:07 ± 4 h 03 min	04:07 ± 1 h	°	-	−1 h 45 min ± 4 h 11 min	−4 h 08 min ± 4 h 55 min	-
		Mean	0.53 ± 0.06	0.52 ± 0.07	-	0.49 ± 0.09	0.41 ± 0.05	-	*	−6.7 ± 11.7	−20.1 ± 8.5	°

The variation of parameters is calculated as the difference between restricted and control values. Non parametric tests were performed with or without pairing (Wilcoxon and Mann-Whitney, respectively). ° <0.1, * < 0.05, ** < 0.01, *** < 0.001.

**Table 2:** Analysis of deviance table (type II Wald Chi-square test) (***< 0.001; ** < 0.01; *<0.05; °< 0.1).

**Parameter**			**Body mass**		**Sex**		**Caloric restriction**		**Interaction**	
			***Chisq***	***p-value***	***Chisq***	***p-value***	***Chisq***	***p-value***	***Chisq***	***p-value***
Creatinine (mg.ml^−1^)			3.56	°	0.009	-	19.96	***	0.002	-
Cortisol (ng.mgCreat. ^−1^) *(poisson)*			153.71	***	0.07	**-**	299.47	***	61.78	***
8-OHdG (ng.mgCreat. ^−1^)			NS		13.26	***	743.64	***	0.01	-
*(poisson)*			Age effect : Chisq =7.41(**)							
Thiols (μmol.mgProt. ^−1^)			NS		0.55	-	4,01	*	6,69	**
GPx (U.mgProt. ^−1^)			NS		2.76	°	0.02	-	7.80	**
17beta-Estradiol (pg.mgCreat. ^−1^)			NS		5.11	*	30.28	***	1.52	-
Testosterone (ng.mgCreat. ^−1^) *(poisson)*			9.63	**	10.71	**	0.99	-	18.23	***
Glycaemia (mg.dl^−1^) *(poisson)*			NS		12.54	***	0.51	-	5.69	*
Body Mass (g)					7.55	**	140.24	***	2.37	-
Body Mass loss (g) *(lm)*			Initial body mass		0.052	-				
			F-value= 10.9 **							
Oxydative coupling rate (mlO_2_.min^−1^)			NS		3.83	°	0.43	-	6.23	*
Mitochondrial reserve capacity (mlO_2_.min^−1^)			NS		2.61	°	5.57	*	1.29	-
Mt/Nu DNA Ratio			NS		4.03	*	2,86	°	0.08	-
RER	*NIGHT*	Max	NS		0.01	-	11.10	***	6.77	**
		hMax	9.40		0.37	-	0.55	-	0.62	-
		Mean	NS		0.70	-	38.34	***	7.40	**
	*DAY*	Min	NS		1.98	-	0.31	-	1.01	-
		hMin	NS		1.81	-	10.18	**	0.89	-
VO2 (ml.kg^−1^.h^−1^)	*NIGHT*	Max	NS		0.34	-	0.33	-	0.01	-
		hMax	339.95	***	0.36	-	22.57	***	395.18	***
		Mean			3.13	°	0.42	-	5.25	*
	*DAY*	Min	NS		2.61	-	9.22	**	21.13	***
		hMin	NS		0.07	-	33.81	***	3.05	°
		Mean			0.003	-	0.013	-	7.37	**
EE (kcal.h^−1^)	*NIGHT*	Max	NS		6.57	*	7.02	**	0.36	-
		hMax	NS		0.02	-	9.02	**	334.48	***
		Mean	NS		8.28	**	20.07	***	1.01	-
	*DAY*	Min	NS		9.03	**	22.32	***	8.4	**
		hMin	NS		8.19	**	62.64	***	15.73	***
		Mean	NS		1.69	-	18.65	***	4.93	*

Linear or generalized mixed effect models with random individual effect: Parameter ~ Body mass + Diet*Sex + (1|Indiv). Body mass was implemented in models when applicable. For non-Gaussian parameters, generalized mixed models with random effects were used when supported by the data sets (family “poisson” mentioned when applicable). A simple linear model (lm) was built to test the effect of sex on body mass loss. Models did not converge for a few parameters (T4 and GlcP); for non-parametric analysis, see Table 1.

## Results

### Caloric Restriction Induced a Stress Response in Both Females and Males

**Figure 1:**
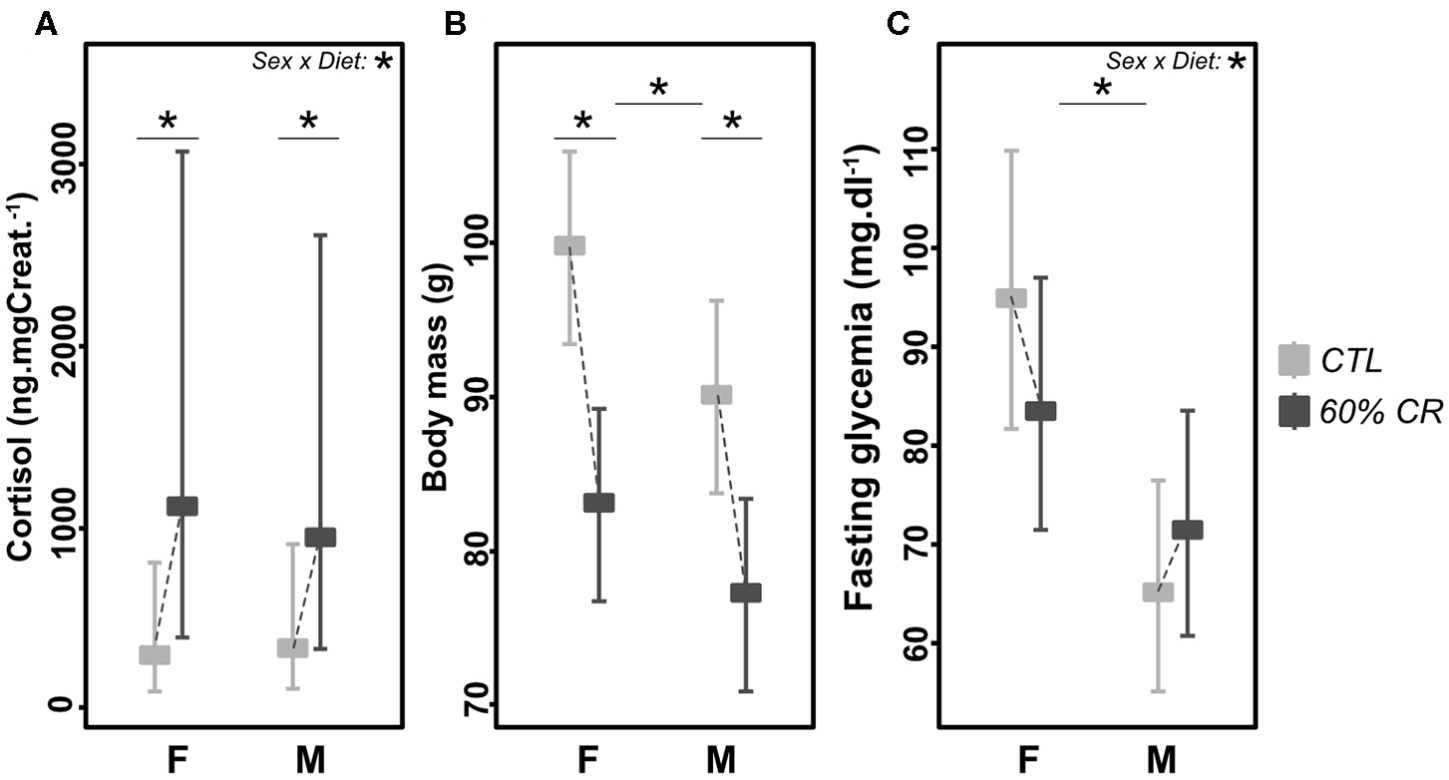
Levels of **(A)** cortisol (ng*mg Creat.^−1^), **(B)** body mass (g), and **(C)** fasting glycaemia (md*dl^−1^) exhibited by female **(F)** and male **(M)** mouse lemurs before (CTL, light gray) and after (60% CR, dark gray) a 2-weeks exposure to a 60% caloric restriction. Data are given as least square means with 95% confidence intervals. Differences within sex groups (effect of CR, lower bars) and between sex groups (effect of sex, upper bars) are represented, as well as the significance of the interaction of Sex*Diet (CTL or CR). *< 0.05.

CR had a significant impact on cortisol production in both sexes (Figure 1A), as the plasma concentration of cortisol increased drastically after treatment (Chisq= 299, *p* < 0.001), with a higher response in females than males (+87.3 ± 142.4% vs. +43.5 ± 34.6%, Chisq= 62, *p* < 0.001; Table 2). One of the first expected effects of CR is the loss of body mass (BM). Although females began the experiment with a higher BM than males (99.7 ± 3.8 g vs. 90.0 ± 6.6 g, *p* < 0.05; Figure 1B), CR induced similar BM loss in both females and males (−16.7 ± 4.3% of the initial BM for females and −14.2 ± 4.3% of the initial BM for males, *p* > 0.05, Table 1) though taking into account the significant effect of the initial BM on BM loss (*p* < 0.01). Concomitantly with BM levels, females maintained a higher glycaemia than males during the experiment under control (95 ± 32 mg.dL^−1^ in females and 65 ± 9 mg.dL^−1^ in males, *p* < 0.001) and CR (84 ± 18 mg.dL^−1^ in females and 72 ± 11 mg.dL^−1^ in males, *p* > 0.05) conditions. However, the effect of CR on glycaemia differed as a function of sex (Figure 1C, *p* < 0.05; Table 2), with no change or very modest decrease in females (−1 ± 51% of the initial glycaemia) and a mean increase in males (+12 ± 26%).

### CR Did Not Induce the Same Metabolic Adjustments in Males and Females

**Figure 2:**
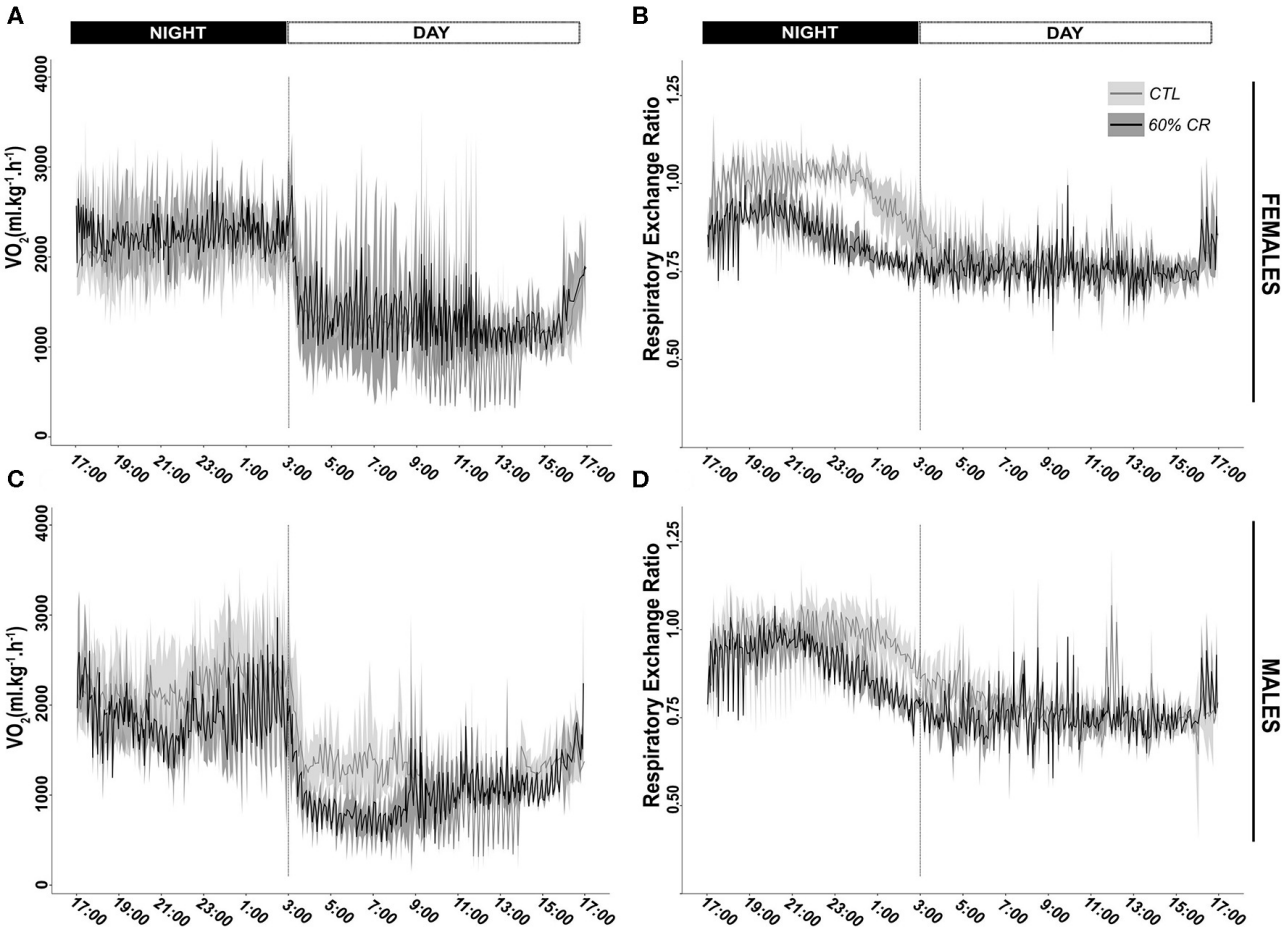
Daily variations (mean ± sd) of oxygen consumption (VO_2_ in ml*kg^−1*^h^−1^) and Respiratory Exchange Ratio (RER) measured in female (**A,B**, respectively) and male (**C,D**, respectively) mouse lemurs before (CTL, light gray) and after (60% CR, dark gray) a 2-weeks exposure to a 60% caloric restriction.

**Figure 3:**
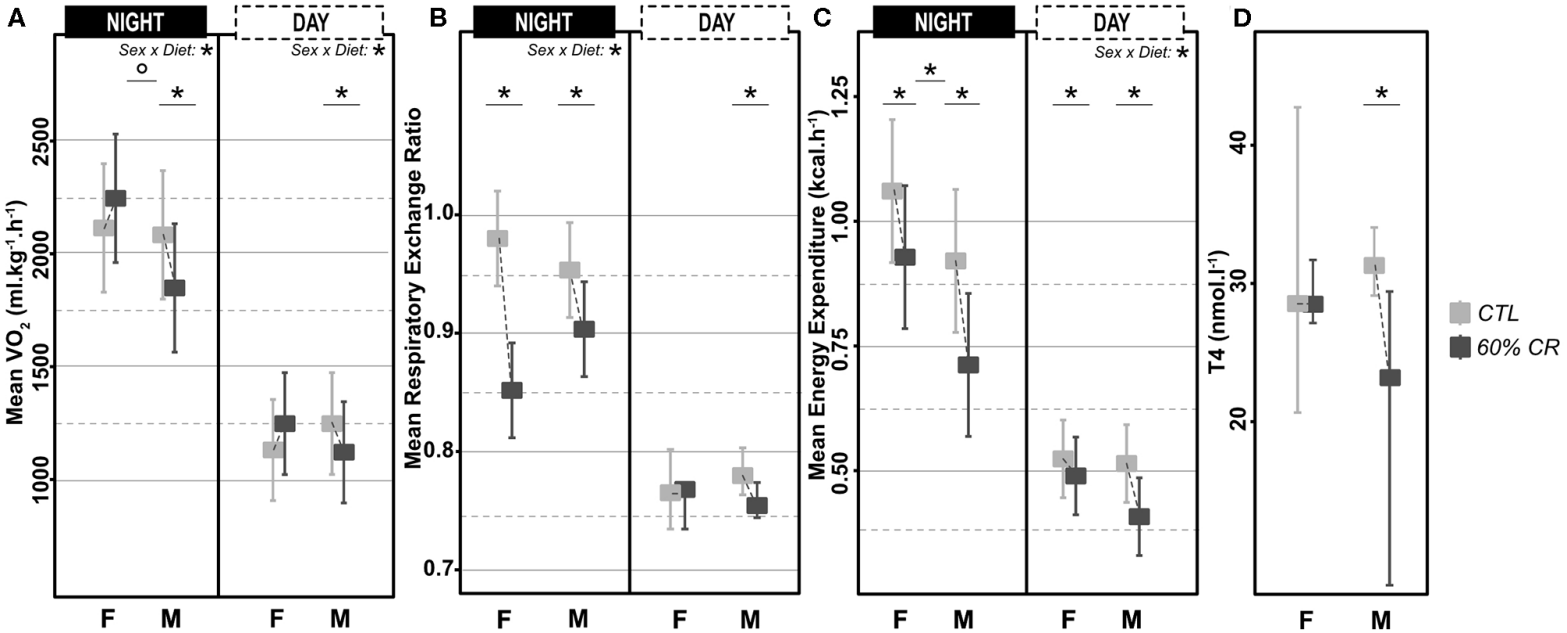
Levels of **(A)** mean oxygen consumption (VO_2_ in ml*kg^−1*^h^−1^), **(B)** Respiratory Exchange Ratio (RER), **(C)** mean Energy Expenditure (in kcal*h^−1^) measured during the night (active phase) and the day (resting phase) in female (F) and male (M) mouse lemurs before (light gray) and after (dark gray) a 2-week exposure to a 60% caloric restriction. Thyroxinemia (T4, in nmol.l-1, **D**) was measured in the same animals during the resting period only. Boxes indicate least square means and error bars represent the 95% confidence interval, except for T4, which is represented as median and total range. Significant differences within sex groups (effect of CR, lower bars) and between sex groups (effect of sex, upper bars) are represented. *< 0.05.

**Figure 4:**
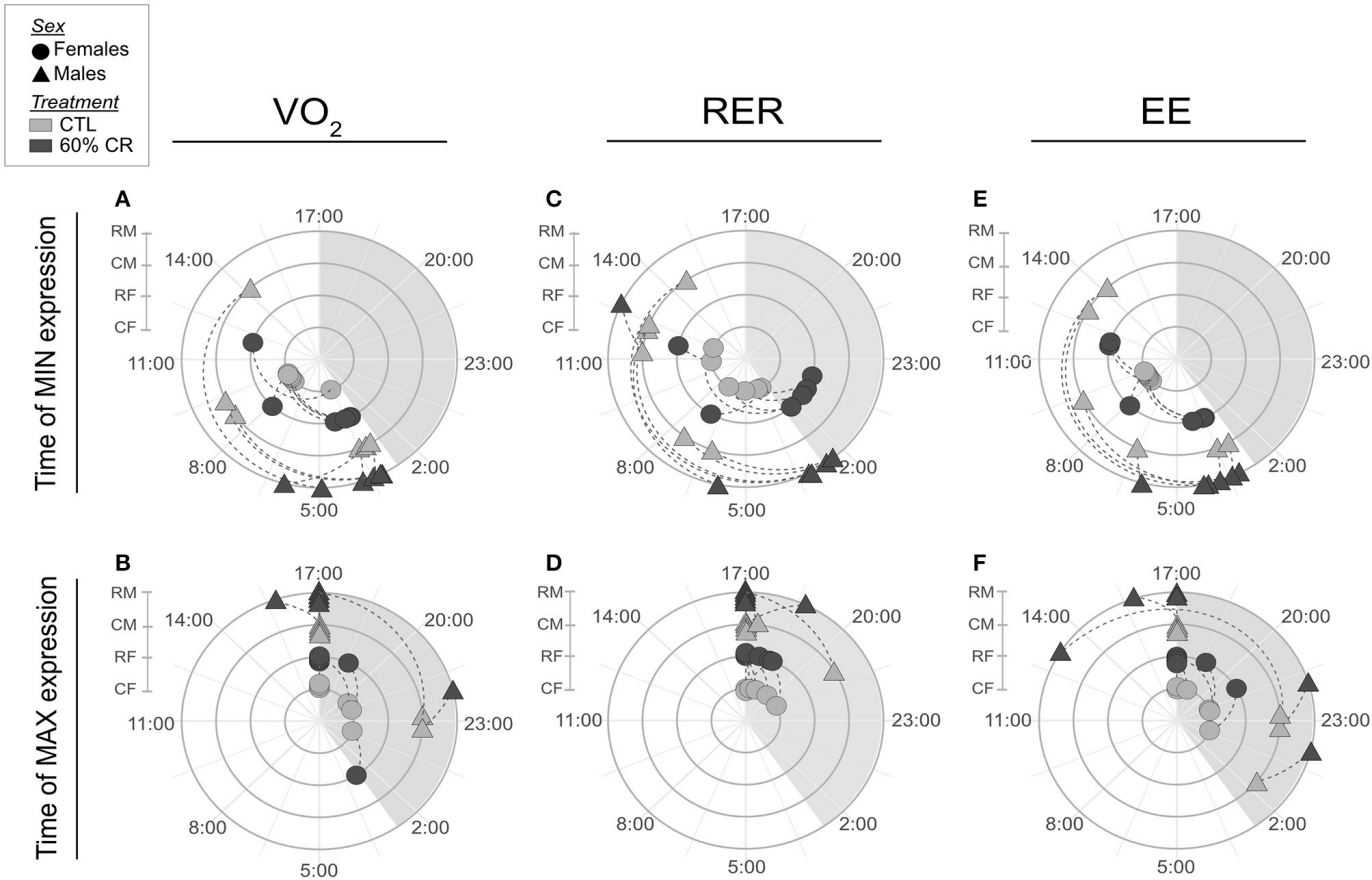
Polar graphics representing averaged time (in hh:mm) when minimum and maximum VO_2_ (**A,B**, respectively), RER **(C,D)**, and EE **(E,F)** were expressed over a 24 h period. Female mouse lemurs are shown as circles, and male as triangles. CTL animals are in ligth gray, CR in dark gray. Each experimental group is represented on its own concentric circle: RM (CR males), CM (CTL males), RF (CR females) and CF (CTL females). Time shift is represented by a dashed line for each individual. The shaded phase between 17:00 and 3:00 is the dark phase of the day, when the lights are off.

Mean oxygen consumption (VO_2_, ml.kg.h^−1^) was reduced after CR in males only, during both active (night: −10.2 ± 9.6%) and resting (day: −9.4 ± 8.8%) periods (Figures 2A,C, 3A; Table 2). Conversely, VO_2_ was increased after CR in females (+7.7 ± 15.9% during night and +9.6 ± 14.9% during day), this being significant during the day only (*p* < 0.05; Tables 1, 2). This sex-specific response to caloric restriction was accompanied by a modification of the temporal pattern of daily metabolic profiles (Figure 4). Indeed, the lower values of VO_2_ were expressed earlier during the day after CR (i.e., resting phase) in both sexes (−2 h 19 min ± 5 h 25 min for females, −2 h 53 min ± 4 h 42 min for males), with an advanced time of maximum VO_2_ during the night (i.e., active phase) (−41 min ± 2 h 19 min for females, −1 h 22 min ± 2 h 15 min for males) that was significantly sex-specific (*p* < 0.001; Table 2).

During the night in CTL situation (Figures 2B,D, 3B), mean values of RER were close to 1 for both males and females (0.99 ± 0.08 for females and 0.96 ± 0.1 for males), reflecting their feeding behavior and indicating their nocturnal carbohydrate-depending metabolism. Under caloric restriction, mean nocturnal RER was decreased in both males and females (0.85 ± 0.076 for females vs. 0.90 ± 0.085 for males) indicating that animals relied less on carbs after feeding under CR as compared to the CTL situation. The daily profiles of RER (Figure 2B) clearly showed that animals had to switch from carb use to lipid oxidation earlier during the night under CR. Even though both sexes followed the same trend, the decrease in nocturnal mean RER was greater in females than in males (−13.0 ± 3.7% for females and −4.9 ± 5.9% for males, *p* < 0.05). However, males but not females showed a significant decrease in their mean RER during the day (−3.6 ± 1.8%), while females' response to CR was very heterogeneous (−0.9 ± 5.2%), which led to a significant sex^*^Diet effect (*p* < 0.01). The time of minimum and maximum expressions of RER during day and night (Figures 4C,D respectively), were significantly advanced after CR during the day (*p* < 0.01, Table 2), although it was not significant during the night and no sex effect could be pointed out (Tables 1, 2).

As a product of VO_2_ and RER, Energy Expenditure (EE, in kcal.hr^−1^) was reduced in response to caloric restriction in both sexes during the night (−11.7 ± 10.8% for females and −21.2 ± 11.6% for males) when animals were active and fed themselves (Figure 3C). The nocturnal mean levels of EE were however higher in females than in males under both CTL (1.06 ± 0.15 kcal.hr^−1^ for females and 0.92 ± 0.16 kcal.hr^−1^ for males) and CR conditions (0.93 ± 0.11 kcal.hr^−1^ vs. 0.71 ± 0.06 kcal.hr^−1^ in females and males, respectively). During the day the mean energy expenditure was decreased by caloric restriction in all animals, but at a greater extent in males (−6.7 ± 11.7% for females and −20.1 ± 8.5% for males). This adjustment in EE levels was accompanied by a temporal shift in the daily profiles of energy expenditure, which was much more pronounced in males than females (Figures 4E,F). Indeed, the shift for an earlier energetic saving was much greater in males under caloric restriction (−1 h 45 min ± 4 h 11 min for females vs. −4 h 08 min ± 4 h 55 min for males, *p* < 0.001; –). During the night, the level of maximum energy expenditure was also shifted due to CR, again at a greater extent in males (effect of sex in CR response *p* < 0.001; Figure 4 and Table 2). Overall, males seem to respond more intensely and temporally to caloric restriction than females by decreasing their overall energy expenditure.

Thyroid hormones confirmed a sex-specific variation in metabolic activity due to caloric restriction (Figure 3D), with a significant decreased concentration of T4 in males only (−6.4 ± 46.6% of in females vs. −35.6 ± 27.4% in males; Tables 1, 2).

### Mitochondrial Activity Responds Differently to Caloric Restriction Between Sexes

**Figure 5:**
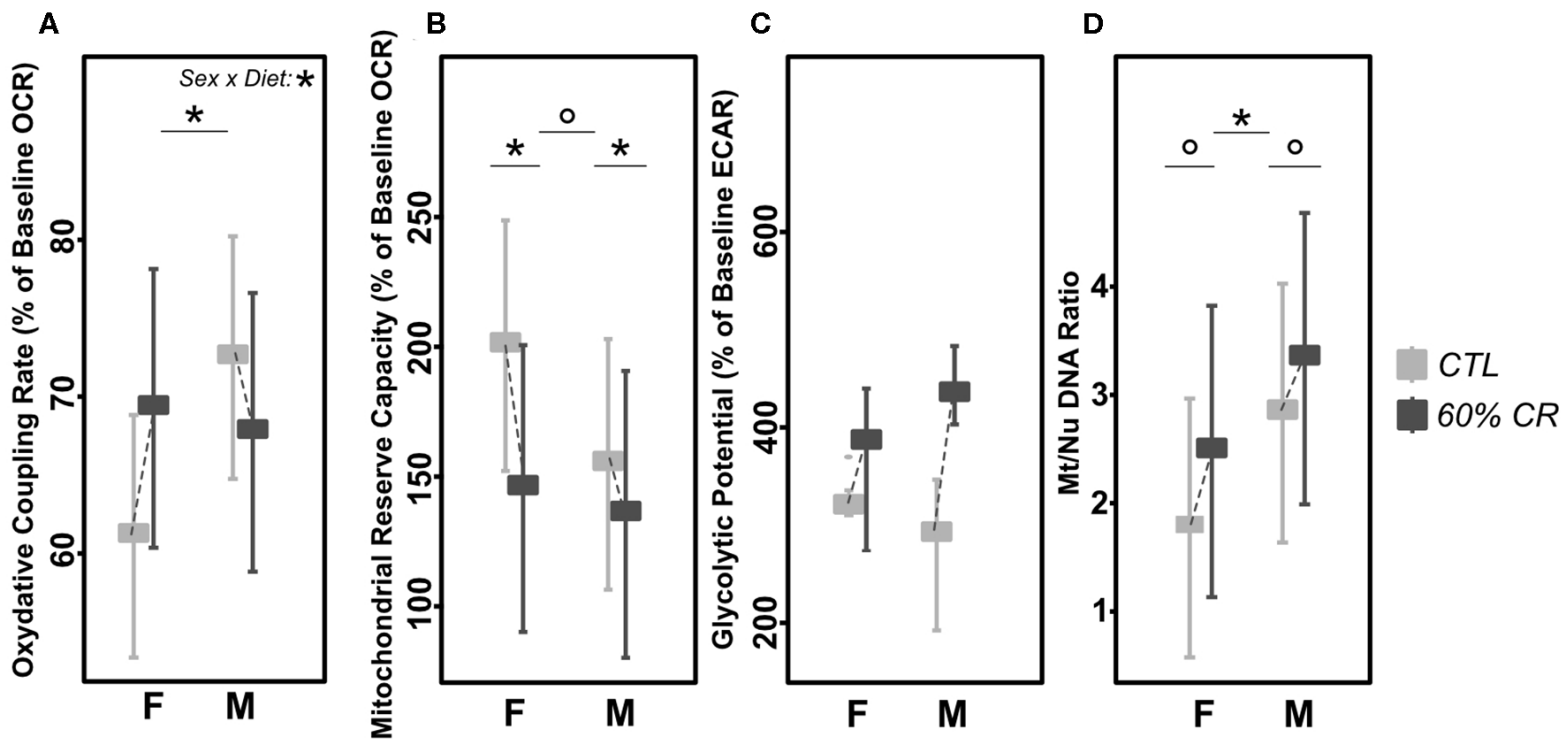
Levels of **(A)** mitochondrial oxidative coupling rate (% of baseline OCR), **(B)** Mitochondrial reserve capacity (% of baseline OCR), **(C)** Glycolytic Potential (% of baseline ECAR), and **(D)** Mt/Nu DNA ratios measured in cultured fibroblasts from female (F) and male (M) mouse lemurs before (CTL, light gray) and after (60% CR, dark gray) a 2-week exposure to a 60% caloric restriction. Boxes indicate least square means and error bars represent the 95% confidence interval, except for GlcP (median and total range). Significant differences within sex groups (effect of CR, lower bars) and between sex groups (effect of sex, upper bars) are represented, as well as the significance of the interaction of Sex*Diet (CTL or CR). *< 0.05; ° < 0.1.

**Figure 6:**
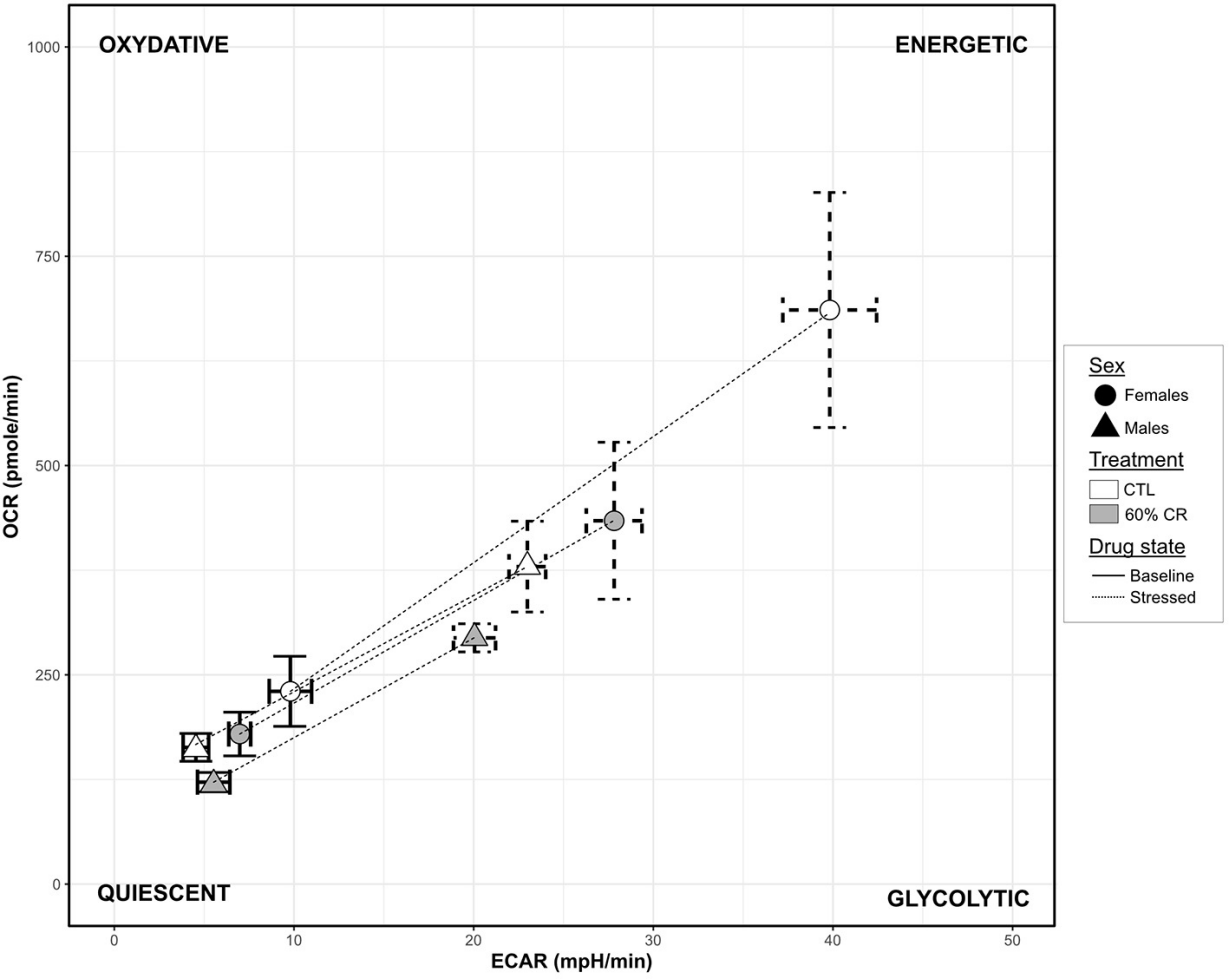
Graphic representation of the cellular metabolic potential measured in cultured fibroblasts from female (circles) and male (triangles) mouse lemurs before (CTL, white) and after (60% CR, gray) a 2-week exposure to a 60% caloric restriction. Metabolic Potentials (mean + sd) are represented by dashed lines between two cell energy phenotypes: “Basal OCR/ECAR” (sd in solid lines) and “Stressed OCR/ECAR” (sd in dashed lines; determined under FCCP conditions, chemically mimicking an induced energy demand). The 2-dimension description of the metabolic potential informs on the general capacity of the cells to meet an energy demand either by glycolysis (“Glycolytic” phenotype), oxidative respiration (“Oxydative” phenotype) or both (“Energetic” phenotype).

Males' mitochondrial oxidative coupling rate (OxCR) measured from cultured fibroblasts was significantly higher than females' under control conditions (Figure 5A). OxCR's response to CR varied according to sex (sex^*^Diet, *p* < 0.05; Table 2), as it increased in females after caloric restriction (+9.77 ± 7.78% of baseline OCR), while a slight decrease was observed in males (−2.97± 12.06% of baseline OCR), thus leveling females' values to those of males' after CR. This rather opposite response of oxidative rate to CR between sexes did not prevent the decrease of mitochondrial reserve capacity (MtRC) in each case (Figure 5B), which was reduced at the same extent due to CR, although females began the experiment with a higher MtRC than males (202.4 ± 45.9% of baseline OCR vs. 152.0 ± 21.8% of baseline OCR, p < 0.1; Table 1). Glycolytic potentials (“GlcP,” Figure 5C) increased under CR showing the enhanced capacities of the cells to use glycolysis under food restriction (+35.3 ± 36.3% of baseline ECAR for females and +44.7 ± 101.0% of baseline ECAR for males, *p* < 0.05; Table 2). From a qualitative assessment, the OCR/ECAR quotients clearly showed a shift from a quiescent phenotype at baseline to a more energetic phenotype after FCCP exposure in all experimental groups, not favoring either the glycolytic or oxidative pathway (Figure 6). Also, caloric restriction reduced the metabolic potential (defined as the difference between the baseline and the stressed condition) of both females and males, although this reduction seemed to be greater in females than in males. However, females consistently showed a greater potential than males, both before and after CR (Figure 6). Mt/Nu DNA ratios (Figure 5D) increased after CR in both sexes (+ 3 ± 26 in females, +60.1 ± 30 in males, *p* < 0.1; Table 1) but males had an overall higher ratio than females before and after CR (*p* < 0.05, Table 2).

### CR Affected the Oxidative Status, Through Different Mechanisms in Males and Females

**Figure 7:**
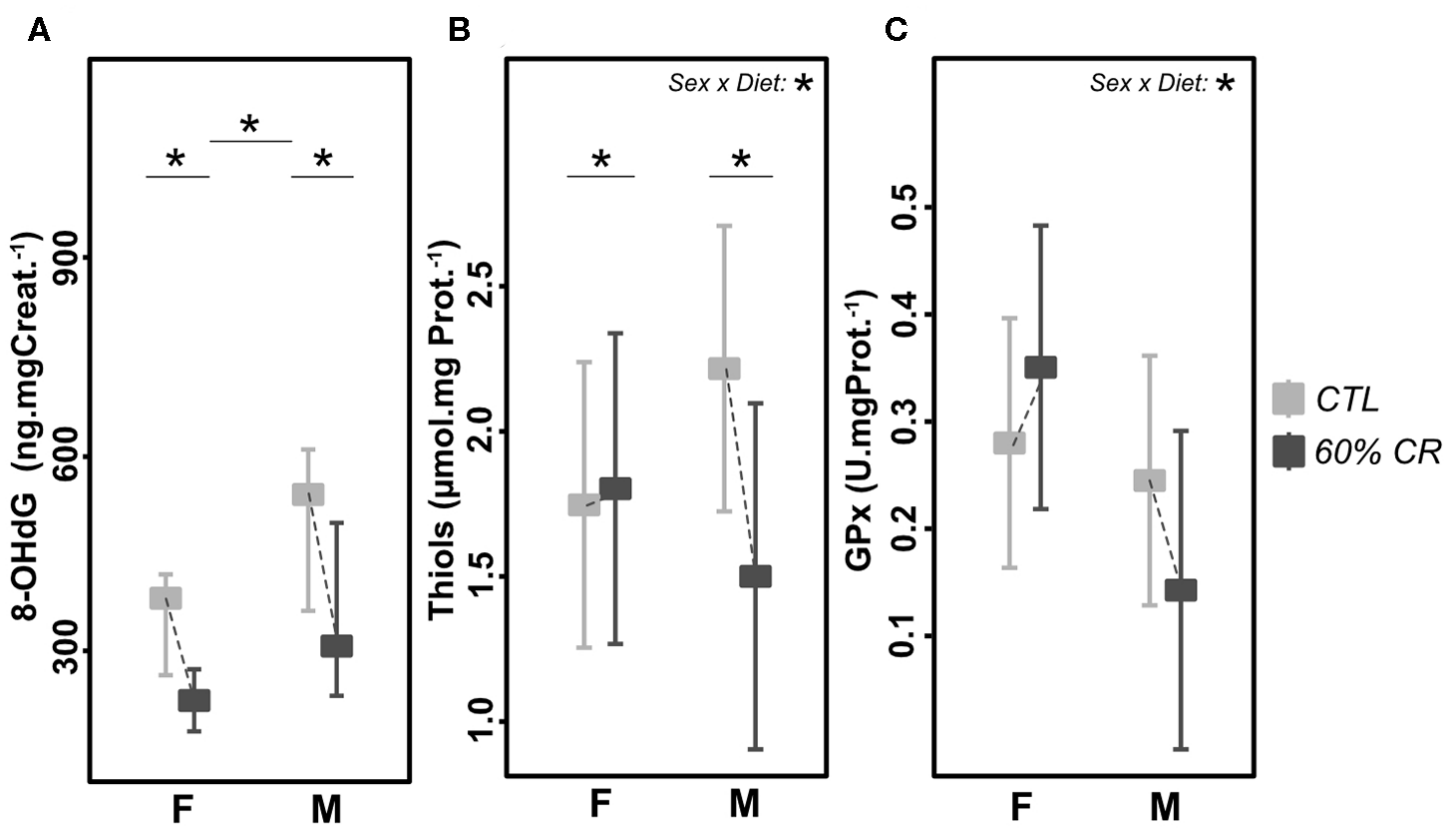
Levels of **(A)** 8-OHdG (ng*mg Creat.^−1*^g.^−1^), **(B)** Thiols (μmol*mg Prot.^−1^), and **(C)** Gluthatione Peroxydase (GPx, U*mg Prot.^−1^) measured in female **(F)** and male **(M)** mouse lemurs before (CTL, light gray) and after (60% CR, dark gray) a 2-week exposure to a 60% caloric restriction. Boxes indicate least square means and error bars represent the 95% confidence interval, except for 8-OHdG (median and total range). Significant differences within sex groups (effect of CR, lower bars) and between sex groups (effect of sex, upper bars) are represented, as well as the significance of the interaction of Sex*Diet (CTL or CR). *< 0.05.

Oxidative DNA-damage (8-OHdG, Figure 7A) showed a higher overall concentration in males than females (387.4 ± 93.0 for females vs. 591.0 ± 262.4 ng.mgCreat.^−1^ for males under CTL diet, *p* < 0.1; 214.1 ± 36.3 for females vs. 324.6 ± 110.8 ng.mgCreat.^−1^ for males after CR), but was decreased after caloric restriction at the same extent in both sexes (−42.4 ± 14.9% for females and −42.7 ± 12.8% for males, *p* > 0.1). However, thiols (Figure 7B) and glutathione peroxidase (GPx, Figure 7C), both markers of anti-oxidant activity, followed the same trend in response to CR, though with opposite directions between females and males (significant interaction effect, *p* < 0.01 in both parameters). Indeed, CR induced an increase in thiols (+4.9 ± 187.1%, *p* > 0.1) and in GPx (+28.85 ± 49.06%, *p* > 0.1) in females, but a decrease in males (thiols: −29.8 ± 44.8%, *p* < 0.1; GPx: −48.08 ± 19.71%, *p* > 0.1).

### Caloric Restriction-Induced Modulation of Sexual Hormones

**Figure 8:**
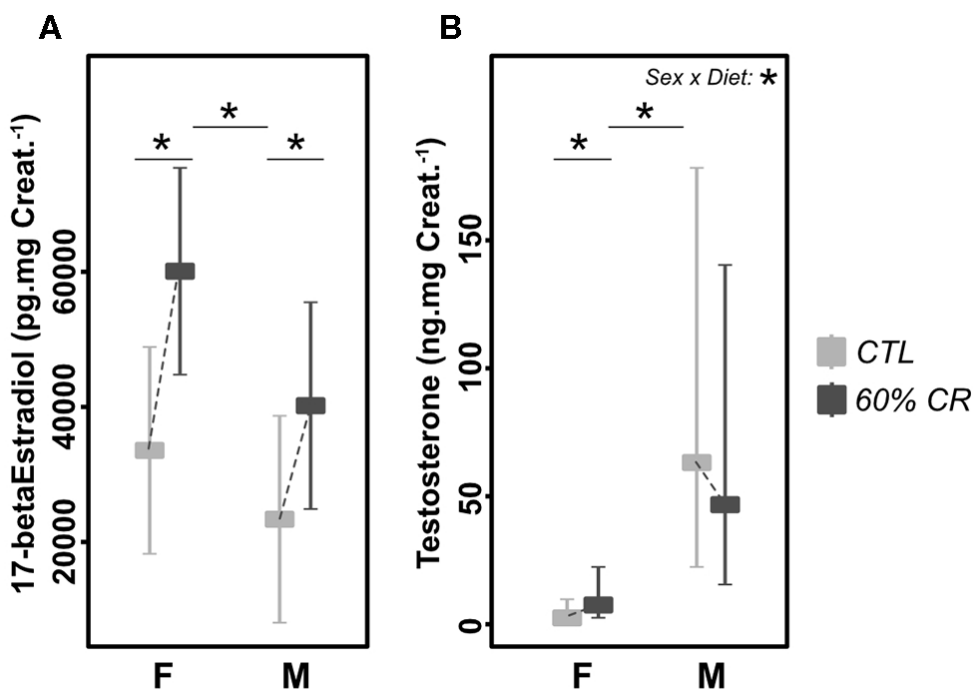
Levels of **(A)** 17-beta Estradiol (pg.mg Creat.^−1^) and **(B)** Testosterone (ng.mg Creat.^−1^) measured in female **(F)** and male **(M)** mouse lemurs before (CTL, light gray) and after (60% CR, dark gray) a 2-week exposure to a 60% caloric restriction. Boxes indicate least square means and error bars represent the 95% confidence interval. Significant differences within sex groups (effect of CR, lower bars) and between sex groups (effect of sex, upper bars) are represented, as well as the significance of the interaction of Sex*Diet (CTL or CR). *< 0.05.

Urinary estradiol was significantly higher in females as compared to males during the experiment (Chisq= 5.11, *p* < 0.05; Figure 8A and Table 2). However, CR induced an increase in both sexes (Chisq = 30.28, *p* < 0.001; Table 2). In parallel, testosterone was very low in females as compared to males (2.8 ± 1.9 ng.mgCreat.^−1^ for females and 64.1 ± 31.1 ng.mgCreat.^−1^ for males in CTL situation, Figure 8B), and exposure to CR induced a significantly increased concentration in females only (+677.1 ± 947.8% in females against +32.8 ± 62.6% in males, <0.05; Table 1), though female levels remained much lower than males' after CR.

### Integrative Analyses Confirmed an Effect of Sex in Response to CR

**Figure 9:**
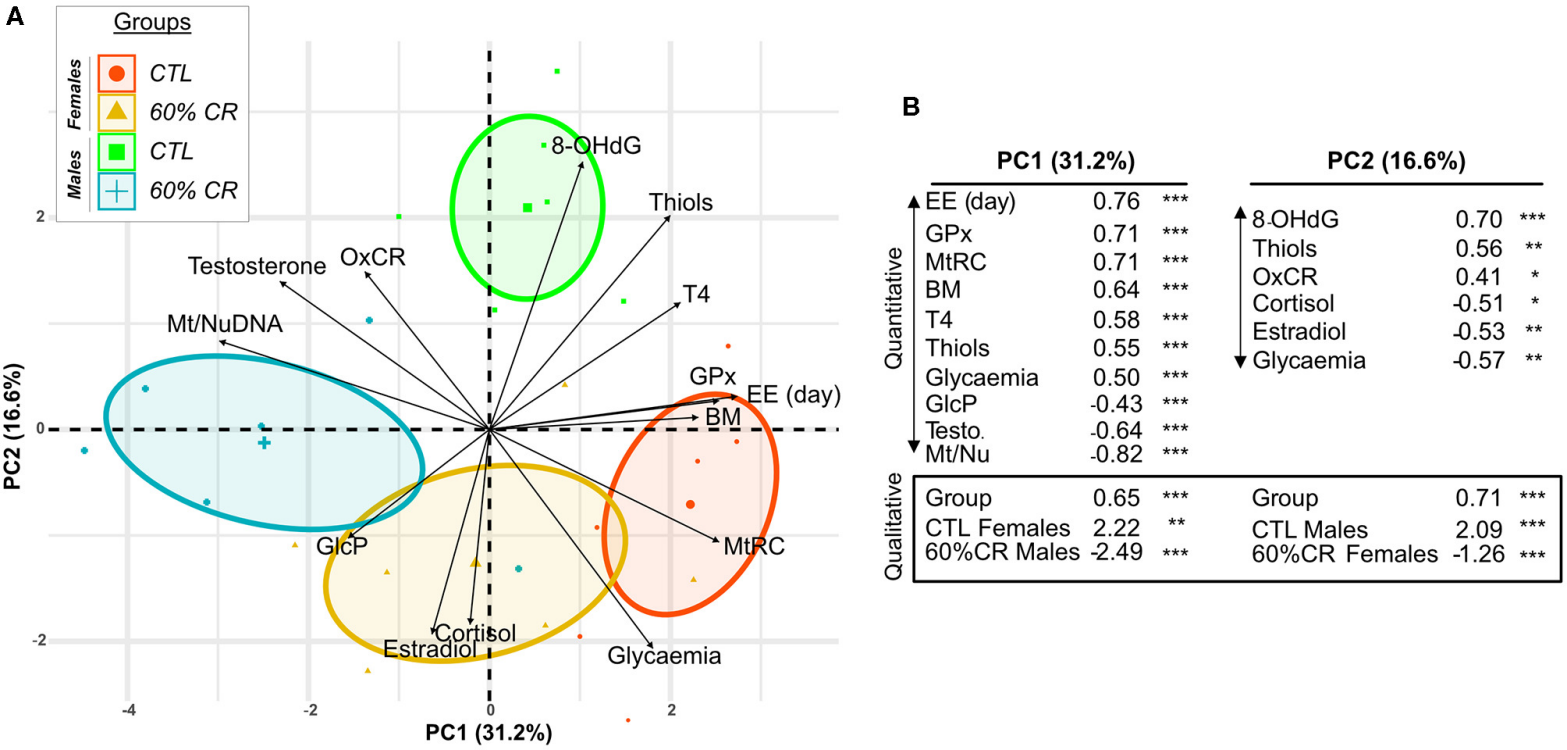
Principal Component Analysis discriminating female and male mouse lemurs before (CTL, red and green for females and males, respectively) and after (60% CR, orange and blue for females and males, respectively) a 2-week exposure to a 60% caloric restriction. Parameters indicative of energy balance (Body mass “BM”), metabolic activity (Cortisol “Cortisol,” Thyroxin “T4,” Mean EE over day “EE (day),” mitochondrial respiration (Oxydative coupling rate “OxCR”, Mitochondrial Reserve Capacity “MtRC,” Glycolytic Potential “GlcP”), Oxidative status (8-OHdG “8-OHdG,” Thiols “Thiols,” Glutathione Peroxydase “GPx”), sexual hormones (Estradiol “Estradiol,” Testosterone “Testosterone”) were included in the analysis. Individual plots are shown in **(A)** and grouped into sex*Diet. The contribution of each parameter is also represented by black arrows displaying the “Variable factor map.” Principal Component 1 (PC1) indicates ~31% of variability and Principal Component 2 (PC2) ~17%. **(B)** Table of correlation coefficients and corresponding *p-*values shows only the variables significantly contributing to the two first principal components of the analysis (Quantitative) and the discrimination of qualitative variables: CTL Females, 60%CR Females, CTL Males, 60%CR Males. *< 0.05, **< 0.01, ***< 0.001.

General PCA analysis (Figure 9) showed a clear discrimination between the 4 experimental groups around the 2 first principal components (PCs), which explained ~50% of the variation in the dataset. First, the analysis demonstrates that males and females started the experiment with different physiological states. PC1 explained about 31% of the variation and significantly discriminated CTL females (*p* < 0.01) and CR males (*p* < 0.001) from the rest of the individuals. The fattest animals (CTL females) were also the ones who had the biggest mitochondrial reserve capacity (MtRC) and the highest energy expenditure (EE) during the day. This was accompanied with a higher thyroxinemia and glycaemia than every other animal (Figure 9B). In contrast, CTL males were characterized along PC2 (which explained around 17% of variability) by their levels of oxidative damage, blood thiols and mitochondrial oxidative coupling rate (Figure 9B). Animals that had the highest levels of oxidative DNA damage (CTL males, *p* < 0.001) were the ones presenting the highest concentrations of thiols and also secreting the lowest cortisol. Caloric restriction induced contrasted effects between males and females along the 2 PCs. Thus, the effect of CR in females was mostly explained by parameters representative of mitochondrial respiration (GlcP, OxCR and the ratio Mt/Nu DNA) as well as estradiol and cortisol levels (Figure S1B), some of which (estradiol and cortisol) significantly contributed to discriminate the CR females from the other animals (Figure 9). Correlations networks (Figure S2) further confirmed strong differences between males and females in the relationship between metabolism and oxidative status. Indeed, energy-related parameters (energy expenditure, VO2, RER; thyroxin levels) were clearly positively correlated with oxidative damage and anti-oxidant activity in males. Such interaction was not evidenced in females, markers of anti-oxidant activity being even negatively correlated to energy expenditure before and after CR.

## Discussion

Facing food shortage after reproductive investment might be very challenging, though females and males might respond unequally to such unpredictable stress. We tackled this question by comparing the response of female and male mouse lemurs to a 2-weeks 60% caloric restriction (CR) applied at the end of the reproductive season. Our results confirmed that CR triggers a different response between males and females, characterized by sex-specific metabolic, anti-oxidant and mitochondrial regulations, which provides experimental evidence for the necessity to consider sex as a factor for population dynamics in climate change models.

### Females Completed Their Summer-Like Season in Better Body Condition Than Males

At the end of summer, many different biological and behavioral events have specifically distinguished males and females, and this could introduce a bias when facing environmental perturbations. With the direct observation that the male sample in this study had an initial lower body mass (BM) compared to females, as it is also the case in Brunoy's breeding colony during this time of the life cycle, we kept in mind that initial BM could influence each sex response to CR differently. In this respect, initial BM was always included in the statistical models and was either left or removed in case of significant or non-significant weight, respectively. However, the mean initial BM in each group was far from representing the poor body condition or “exhaustion” that we could expect to find in wild populations (as animals from both sexes have been found to weight up to ~60 g during the transition to winter, Schmid and Kappeler, 1998). Also, the sex-gap of this particular parameter, although statistically significant, is very limited compared to the extreme BM variations one individual can display throughout the year (with an amplitude of ~50% of the mean BM variation over 1 year, Perret et al., 1998). Nevertheless, even after spending summer under the “favorable” conditions of Brunoy's colony, with regular and predictable food intake, our results confirmed that CR triggers different mechanisms between males and females at the end of the summer-like period, characterized by sex-specific metabolic, anti-oxidant and mitochondrial properties. In this line, results from multivariate analyses confirmed that BM was not the primary contributor of the sexual differences observed in response to CR.

### Caloric Restriction Was Perceived as a Stressor During the Summer-Like Period

One remarkable result was the significant and consistent cortisol increase in urine samples after CR in both females and males, which could point out a stressed physiological status (for review, see Breuner et al., 2013). The effect of CR on cortisol concentrations seems to depend on its intensity and duration; in a recent meta-analysis, only fasting (from 2.5 to 6 days) was responsible for an increase in cortisol in human patients, while a 60% CR between 1 to 28 days did not (Nakamura et al., 2016). Previous work in rats showed a dose-dependent response of the increase in serum corticosterone after 3 weeks of CR (Levay et al., 2010). In mouse lemurs, a 60% CR applied to 24 females in winter-like period, as well as a chronic food shortage of 40% followed by a 80% acute CR in 6 individuals induced no urinary cortisol effect as well (Canale and Henry, 2011). The present experimental study was actually the first to test the effect of CR at this advanced time of the summer season, which could explain the difference with the results mentioned above. We found that a 60% CR undergone after the reproductive effort is therefore felt as a stress for these highly flexible primates, thus confirming the unexpected nature of such food shortage during a usually well supplied season (Dammhahn and Kappeler, 2008). In contrast, mouse lemurs are physiologically well-adapted to food shortage during winter (a dry period with low food availability), which thus triggers low stress response but induce hypometabolism states instead (Giroud et al., 2008; Vuarin et al., 2015).

### Males and Females Show a Similar Energy Balance but Different Metabolic Responses to CR

As lemurs went through their energetic challenges of the year related to reproduction—though these challenges seem to be quite moderated for females in captive conditions (Landes et al., 2019)—late summer is considered as a period of nutritive abundance and possibly fattening in healthy natural populations that anticipate the poor season (Radespiel, 2006; Radespiel et al., 2006; Dammhahn and Kappeler, 2008). At the time of the experiment, males had undergone their reproductive investment and were sexually inactive, although testosterone levels were still high before and after CR. Females had also completed gestation and parental care, and did not exhibit late estrus. Therefore, we considered that male and female mouse lemurs probably shared equivalent energy allocations, although the experimental design did not allow to firmly rule out any confounding factor due to the natural modulations in metabolic activity that might have happened within the 2-weeks duration of the experiment, as animals were their own controls. Nevertheless, mouse lemurs are supposedly stable in their metabolic and reproductive status at this time of the year in captive conditions (Perret and Aujard, 2001). In these conditions, males and females lost similar amount of weight following CR, in total and relative to their initial body mass. The latter had an impact on the amount of weight loss, and thus erased any sex effect in the individuals' reaction to caloric restriction. In the same time, the mitochondrial reserve capacity (MtRC) decreased in both sexes, reflecting the impact of CR on the animals' ability to face new environmental stressors, as the cell's capacity to meet a new energy demand is drastically reduced under impaired caloric intake. Furthermore, MtRC was indeed positively correlated with body mass (Figure 9, Figures S1, S2), which strengthens its interpretation as an energy-balance marker.

However, the underlying metabolic indicators presented high variability between sexes, with an overall diminution in males (decrease in night and day oxygen consumption—VO_2_, Energy Expenditure—EE, Thyroxin parameters, no change for Oxidative Coupling Rate—OxCR) but very contrasted changes in females (mean VO_2_ increased during the day and did not vary with CR during the night, resulting in an unchanged EE during the day and decrease over night; no effect of CR was observed on T4 but OxCR increased). We can thus argue that even if CR was perceived as a stressor by both sexes, and induced similar energy balance, there is conclusively a sex-specific physiological response to this type of stressor at the end of summer. These results suggest that males entered a hypometabolic state, while females did not, particularly during the day when torpor, which is an energy-saving mechanism (Vuarin et al., 2015), occurs. As we used the total body mass to normalize VO_2_ measurements instead of the lean body mass (LBM), which is supposed to be the metabolically active portion of the total weight, the male-female VO_2_ gap after CR is likely to be underestimated. Moreover, in regard to the final energy balance, hypometabolism was not efficient in males and was not followed by a decrease of ATP needs, as OxCR informs on the energy production rates of the cells (Divakaruni et al., 2014). In contrast, females' ATP demand increased in response to CR, but was still significantly lower than in males. Aside from this, we observed an increase in glycolysis potentials (GlcP) after CR in both sexes, meaning that the cells were able to better use glycolysis in case of an extra fuel need under stressed conditions and maximal mitochondrial activity. CR seemed to favor a more direct type of response to environmental stress with increased glycolysis capacities, while MtRC and thus maximal mitochondrial respiratory capacity under stress lowered drastically and could not provide full proficiency. Cellular metabolic potential was decreased after CR showing an altered response toward induced cellular energy demand, but were still—although qualitatively assessed—higher in females. For these cellular-scaled parameters we can still argue on the link between metabolism and fibroblasts, whose response may not represent the one of cells more implied in the regulation of metabolic activity or “metabolic tissues” (as hepatocytes, adipocytes, pancreatic cells or myocytes) (Patel et al., 2014). However, fibroblasts are constitutive of the skin, the barrier between the internal and external environment of an organism, that is capable of integrating many signals which *in fine* impact homeostasis (for review see Slominski et al., 2013). As such, even if fibroblasts are not key actors in the regulation of metabolism, they can still respond to an organism's regulation, and they are known to be up and down-regulated along with key metabolic pathways, as glycolysis and fatty acid oxidation (Zhao et al., 2019). Our method showed good discrimination of mitochondrial activity under CR, sparing the necessity to perform more invasive sampling.

Observations in the little brown bat (*Myotis lucifugus*) converged toward the “thrifty female hypothesis” (TFH; Jonasson and Willis, 2011), in which females display a greater resistance to weight loss than males, as they supposedly experience greater energetic challenges after the dry season, when they face spring reproduction (Key and Ross, 1999). This selection pressure would have driven females to be more efficient in using energy saving mechanisms, or “thrifty phenotypes” (TP), such as torpor, hibernation or fat storage (CZenze et al., 2017; Willis, 2017). The TFH has been originally enunciated to explain sex-variability in the expression of these TP during winter (Humphries et al., 2003; Jonasson and Willis, 2011), but no study has yet explored its generality throughout the year. Indeed, if the expression of a TP in females is dependent on their energy allocation to reproduction, as stated in the TFH, it is worth considering the case of a more constitutive phenomenon, where females' TP could also be expressed after reproductive investment. There is actually growing evidence that females of different species could present physiological abilities compared to males in their response to caloric restriction and energy storage independent of or during gestation (Rodriguez-Cuenca et al., 2002; Valle et al., 2007; Lennox and Goodship, 2008; CZenze et al., 2017). Overall, in the present study, the comparison of physiological responses to CR between males and females did not support the hypothesis that female lemurs might express a more “thrifty” phenotype in late summer, as the energy balance was similar between sexes, and there was a reduced metabolic activity in males. We cannot rule out the fact that females were heavier than males before and after the experiment which might have influenced their response to CR. Indeed, body condition is known to interfere with the use of hypometabolism (Bieber et al., 2014). Given that hypometabolism is associated with adverse effects such as accumulation of molecular oxidative damages (Wei et al., 2018), the “decision” not to initiate it might be more adaptive in females at that season time, according to the fact that they had sufficient reserves to undergo the cost of CR. Whether females would have modified their response to CR with a prolonged stress remains an open question.

### Sex-Specific Oxidative Status Management in Caloric Restriction Conditions

Despite CR was perceived as a stress in regard to the individuals' cortisol increase, urinary oxidative damage to DNA (8-OHdG) decreased in both sexes. It was initially lower in females (and remained lower after CR), which suggests an overall greater oxidative stress in male mouse lemurs at the end of summer. 8-OHdG positively correlated with metabolic activity parameters only in males (mean EE during the day, thyroxin—T4, OxCR see Figure 9, Figures S1B, S2) according to our expectations and the Free radical theory of aging (Harman, 1956). Indeed, intense oxidative metabolism produces reactive oxygen species (ROS) that damage molecular structures, potentially inducing cellular and tissue harm, which eventually contribute to senescence (for review, see Kregel and Zhang, 2007). Anti-oxidative machinery intervenes as a protective mechanism (Pamplona and Costantini, 2011) and could be regulated by metabolic rate as well. Here we observed contrasted relationships between metabolic rate, T4, 8-hydroxy-2'-deoxyguanosine−8-OHdG, glutathione peroxidase and Thiols in males and females mouse lemurs. Indeed, caloric restriction induced concomitant decrease in T4, 8-OHdG and antioxidant machinery in males, thus compelling with the “rate-of-living” theory. In females however no or little change in energy expenditure was observed after CR, which was corroborated by the absence of variation in T4 measurements, while thiols (and GPx to a lesser extent) significantly increased (yet very contrasted amongst the individuals) and 8-OHdG was still reduced, supposedly as a direct consequence of the anti-oxidant activity. Although metabolic activity could be directly implied to some extent in the resorption of oxidative damage (Pamplona and Costantini, 2011) as we observed in males, females seem to activate other pathways, such as the up-regulation of anti-oxidant mechanisms.

We do not know if the intersexual variability in the regulation of oxidative status is a direct effect of sex or is linked to the difference of metabolic rate response induced by CR. As it is discussed in many studies, oxidative stress could be modulated either by food shortage or sexual hormones, and the existence of a sex-specific oxidative balance is debated (Costantini, 2018). Caloric restriction has been robustly demonstrated to improve maximum life span in mammals, including primates (Masoro, 2005; Pifferi et al., 2018) and this effect was correlated with the reduction of oxidative damage accumulation in several experiments (for review, see Sohal and Weindruch, 1996). Recently the protective effect of chronic CR on oxidative stress was diminished in mice that lacked type-3 sirtuin SIRT3, a mitochondria-localized protein which promotes the anti-oxidant properties of type-2 superoxide dismutase SOD2 (Qiu et al., 2010), indicating a major upregulation of the antioxidant machinery during CR. This particular result is in line with our findings in females, but not in males (although the sex of mice was not specified, nor discussed in the above mentioned article). It is also pointed out that estrogen, in contrast to other natural steroids, possess an antioxidant activity because of the phenolic structure in the molecule, and this specificity was assessed *in vitro* in 1987 (Sugioka et al., 1987). Additionally, a positive correlation was observed between estrogen and GPx (Massafra et al., 1998), which supports the hypothesis of an estrogen-dependent regulation of the antioxidant machinery. In rats, castration decreased SOD activity in both sexes, although more intensely in females, and highly increased lipid peroxidation (in a model of myocardial damage) in females, but not in males (Barp et al., 2002). In healthy aged rats finally, females were proven to host lower mitochondrial content than the males (which was supported by our Mt/Nu DNA results), but with a greater differentiation degree and higher activity (Guevara et al., 2009). This was accompanied by a better “oxidative balance” in females, meaning higher respiratory function followed by equal ROS production, but greater antioxidant enzyme activity, and uncoupling proteins (Guevara et al., 2009). All these information converge to a sexual difference in the oxidative status regulation, also in a context of CR, where females are able to lower the damage induced by the perceived stress by upregulating their antioxidant machinery, while males respond by decreasing their metabolic activity, which also decreases ROS production.

As predicted from the above studies, estrogen increased in response to CR in females. The effect was also observed in males, which represents an original result as few studies gather information on the potential role of estrogen as regulator of the oxidative status in males. Our study also provides a first glimpse of *Microcebus murinus* males' estrogen concentrations. We did not find any estrogen-antioxidant correlation however to support the role of the sexual steroids in the regulation of oxidative status, but estradiol levels did correlate negatively with 8-OHdG (see Figure 9, Figures S1, S2).

On the other hand the anti-oxidant effect of testosterone remains unclear, as it has been shown to either depress red blood cell resistance to free radical attack in a male zebra finch model treated with testosterone (Alonso-Alvarez et al., 2007), or decrease ROS production and increase GPx activity and thiols groups content in an *in vitro* human neutrophil culture (Marin et al., 2010). Mouse lemur males expressed more than 20 times the testosterone concentrations of females, yet CR still had a significant impact on females' testosterone levels (Figure 8), while it remained unchanged in males. As testosterone can be metabolized into estradiol and dihydrotestosterone, their respective oxidative/antioxidant properties may be difficult to tease apart. This idea was explored in 2012 in birds (Casagrande et al., 2012), which showed an antioxidant activity of estradiol only in both sexes. The increase in female's testosterone may be due to the necessity of producing higher levels of estrogens, while the “stock” would be already available in males.

### On Sex-Biased Ecological and Evolutionary Perspectives

The final energy balance in response to CR was similar between males and females in the present study. However, the metabolic rate and oxidative damage modulations were significantly different between sexes. Metabolic activity markers of females were not decreased after CR (T4, OxCR, VO_2_) which could illustrate how physiology is adapted to the different biological and behavioral features of sexes in *Microcebus murinus* during this late-summer context, when females still take care of their offspring in nature and form kin-groups to endure winter (Radespiel et al., 2001). Both body mass and mitochondrial reserve capacity were lower in males at the beginning of the experiment (and remained as such afterwards) suggesting that summer would be a more challenging period for them, even in breeding conditions. This conclusion is also supported by the higher levels oxidative damage to DNA.

The major constraint for the fitness of males that could drive sexual selection and dimorphism is thought to be the limited access to females for mating, that would thus favor larger body size or other energetically costly physical features (Darwin, 1874; Emlen and Oring, 1977; Key and Ross, 1999). As opposed to this, females' reproductive success would be constrained –only- by food resources (Trivers, 1972). In case of a monomorphic species, females' energy expenses were demonstrated to exceed that of males, mainly because of gestation and lactation, which costs can be easily monitored (Key and Ross, 1999). The males' specific energy expenditure was actually calculated during the time it took for females to produce an offspring. This particular paper did not take into account the sex-dependent phenological shift of maximal reproductive energy investment that happens in many wild species. In our point of view, spermatogenesis cost is underestimated especially when it happens during harsh environmental conditions, which introduces a bias in the ecological niche framing sex-specific biological energetics. With the tenet of gestation and lactation being energy-consuming processes, females are traditionally thought to be naturally more challenged with a higher fuel need to supply than the males. But with a high spermatogenesis rate, energy allocation for reproduction is probably very costly for male mouse lemurs also (Harcourt et al., 1981; Aslam et al., 2002), as for other species involved in sperm competition.

The ecological “niche” in which animals enter an active metabolic status can be drastically different according to sex (Darwin, 1874; Shine, 1989) and expose males and females to unequal challenges. As males use their fat storage in winter to produce high quantities of gametes in anticipation of the mating season, they are in an unbalanced situation compared to females at the beginning of summer. They thus strongly depend on good environmental conditions and food availability after the main energetic investment, when females can use their energy reserve if an unpredicted food shortage happens during gestation or lactation. Pregnant female bats were recorded to maintain normothermic body temperatures during spring migration and suffer consistently less water loss than males, which entered torpor more frequently (Cryan, 2003). This would tend to dig the gap between males and females on their ability to survive summer, and introduce a “loss of opportunity” to survive in male mouse lemurs. Indeed, field data gather evidence that goes in the direction of a rougher summer for males, with a survival 16% inferior than the one of females' at the beginning of the season (Kraus et al., 2008). It is explained in this paper by the “risky male” theory, where males face dangerous situations during territorial and dispersal behaviors at the early stages of summer during mating. Emphasizing this, male-biased predation seems to be common in nature and is largely documented (see review Christe et al., 2006). This behavioral-based hypothesis does not take into account the energy balance ensuing from physiological mechanisms linked to reproductive activity in males and the rise of testosterone levels, which could be very costly in terms of metabolic activity, immunity and thus fitness (Muehlenbein and Bribiescas, 2005). Moreover, habitat and food resources use is highly influenced by dominance relationships specific of mouse lemurs, which are in favor of females (Radespiel et al., 1998; Hohenbrink et al., 2015). Which of these processes, whether behavioral, physiological or ecological are of primary importance in the field, remains an open question.

## Conclusions

We found evidence of sex-specific regulation of metabolic activity in mouse lemurs submitted to CR at the end of summer, which could compel with their own programmed physiological and behavioral agenda. Contrary to females, males tended to lower their metabolic activity, but they began the experiment with a lower body mass than females and still lost similar amount of weight. Moreover, males and females seem to differ in how they regulate their oxidative status, hormones, and energy expenditure, which appear to differ under both baseline and challenging conditions. In other words, these sex differences might point to various selective pressures across the life stages acting on the physiological machinery of males and females that need to optimize their functions to expression of life-history traits. Although our results came from animal maintained under captive conditions, they suggest that the survival probability until the next season of males is much more dependent than females on the environmental conditions experienced during summer. As the climatic evolution in Madagascar is growing unpredictable with climate change (Canale and Henry, 2010), it is hypothesized that selective pressures would favor individuals with a higher body mass or with greater capacities to store fat (Dewar and Richard, 2007). Whether this could impact sex-ratio of mouse lemur populations may depend on the rapid adaptation of males to food shortage. This type of sex-specific ecological challenge is still poorly discussed and would merit more attention, especially since two-sex models of population dynamics proved that a biased-sex ratio could lead to a rapid collapse in vertebrates (Le Galliard et al., 2005; Grayson et al., 2014).

## Data Availability Statement

The datasets generated for this study are available on request to the corresponding author.

## Ethics Statement

The animal study was reviewed and approved by Cuvier Ethics Committee for the Care and Use of Experimental Animals of the Muséum National d'Histoire Naturelle.

## Author Contributions

AN, FA, and JT designed the work. AN, CR, and LP did the animal work. AN realized all the analytical experiments and statistical analyses. AN and LP did the indirect calorimetry analysis. AN, DC, and JT realized the anti-oxidant activity experiments. AN and LP did the cell culture. AN and J-FR realized the Mt/Nu DNA measurements. AN, FA, LP, and JT analyzed and interpreted the different sets of data. AN, FA, and JT wrote the manuscript. AN, DC, FA, JF-R, and JT reviewed the final manuscript.

## Conflict of Interest

The authors declare that the research was conducted in the absence of any commercial or financial relationships that could be construed as a potential conflict of interest.

## Supplementary Material

Figure S1 Comparison of **(A)** Females and **(B)** Males Principal Component Analysis. Parameters indicative of energy balance (Body mass “BM”), metabolic activity (Cortisol “Cortisol,” Thyroxin “T4,” Mean EE over day “EE (day),” mitochondrial respiration (Oxydative coupling rate “OxCR,” Mitochondrial Reserve Capacity “MtRC,” Glycolytic Potential “GlcP”), Oxidative status (8-OHdG “8-OHdG,” Thiols “Thiols,” Glutathione Peroxydase “GPx”), sexual hormones (Estradiol “Estradiol,” Testosterone “Testosterone”) were included in the analyses. Individual plots are shown in females **(A)** and males **(B)** and grouped into caloric treatment (CTL in red, 60%CR in green). The contribution of each parameter is also represented by black arrows displaying the “Variable factor map”. **(C)** Table of correlation coefficients and corresponding p-values shows only the variables significantly contributing to the two first principal components (PC1 and PC2) of the analysis (Quantitative) and the discrimination of qualitative variables: CTL and 60%CR. ^*^ <0.05, ^**^ <0.01, ^***^ <0.001.

Figure S2 Correlation networks of females and males parameters. Data before and after caloric restriction were all included in the analysis for each parameter and each sex. Each parameter is written in black (Body Mass, Glycaemia, T4, Cortisol, 8OHdG, Thiols, Gpx, Testosterone, Estradiol, mean VO2, EE and RER during day or night). The closer one variable is to another, the higher the relationship. The opposite is true for widely spaced variables. Blue lines represent positive correlations (only correlations from *r* = 0.5 to 1 appear, with *p* < 0.1), while red lines negative ones. The line shade and thickness modelize the strength of the relationship.

## References

[B1] Alonso-Alvarez C. Bertrand S. Faivre B. Chastel O. Sorci G. (2007). Testosterone and oxidative stress: the oxidation handicap hypothesis. Proc. R. Soc. Lond. B Biol. Sci. 274, 819–825. 10.1098/rspb.2006.3764 17251089 PMC2093982

[B2] Aslam H. Schneiders A. Perret M. Weinbauer G. F. Hodges J. K. (2002). Quantitative assessment of testicular germ cell production and kinematic and morphometric parameters of ejaculated spermatozoa in the grey mouse lemur, Microcebus murinus. Reproduction 123, 323–332. 10.1530/rep.0.1230323 11866700

[B3] Barp J. Araujo A. S. R. Fernandes T. R. G. Rigatto K. V. Llesuy S. Bello-Klein A.. (2002). Myocardial antioxidant and oxidative stress changes due to sex hormones. Braz. J. Med. Biol. Res. 35, 1075–1081. 10.1590/S0100-879X2002000900008 12219179

[B4] Bieber C. Lebl K. Stalder G. Geiser F. Ruf T. (2014). Body mass dependent use of hibernation: why not prolong the active season, if they can? Funct. Ecol. 28, 167–177. 10.1111/1365-2435.12173

[B5] Breuner C. W. Delehanty B. Boonstra R. (2013). Evaluating stress in natural populations of vertebrates: total CORT is not good enough. Funct. Ecol. 27, 24–36. 10.1111/1365-2435.12016

[B6] Canale C. I. Henry P. Y. (2010). Adaptive phenotypic plasticity and resilience of vertebrates to increasing climatic unpredictability. Clim. Res. 43, 135–147. 10.3354/cr00897

[B7] Canale C. I. Henry P. Y. (2011). Energetic costs of the immune response and torpor use in a primate. Funct. Ecol. 25, 557–565. 10.1111/j.1365-2435.2010.01815.x

[B8] Canale C. I. Huchard E. Perret M. Henry P. Y. (2012a). Reproductive resilience to food shortage in a small heterothermic primate. PLoS ONE 7:e41477. 10.1371/journal.pone.0041477 22848507 PMC3405090

[B9] Canale C. I. Perret M. Henry P. Y. (2012b). Torpor use during gestation and lactation in a primate. Naturwissenschaften 99, 159–163. 10.1007/s00114-011-0872-2 22159593

[B10] Casagrande S. Costantini D. Groothuis T. G. (2012). Interaction between sexual steroids and immune response in affecting oxidative status of birds. Comp. Biochem. Physiol. Part A Mol. Integr. Physiol. 163, 296–301. 10.1016/j.cbpa.2012.07.018 22885344

[B11] Christe P. Keller L. Roulin A. (2006). The predation cost of being a male: implications for sex-specific rates of ageing. Oikos 114, 381–384. 10.1111/j.2006.0030-1299.15130.x

[B12] Costantini D. (2018). Meta-analysis reveals that reproductive strategies are associated with sexual differences in oxidative balance across vertebrates. Curr. Zool. 64, 1–11. 10.1093/cz/zox002 29492033 PMC5809033

[B13] Costantini D. Wachter B. Melzheimer J. Czirjak G. A. (2017). Socioecological and environmental predictors of physiological stress markers in a threatened feline species. Conserv. Physiol. 5 10.1093/conphys/cox069

[B14] Cryan P. M. (2003). Sex differences in the thermoregulation and evaporative water loss of a heterothermic bat, Lasiurus cinereus, during its spring migration. J. Exp. Biol. 206, 3381–3390. 10.1242/jeb.00574 12939370

[B15] CZenze Z. J. Jonasson K. A. Willis C. K. R. (2017). Thrifty Females, Frisky Males: Winter Energetics of Hibernating Bats from a Cold Climate. Physiol. Biochem. Zool. 90, 502–511. 10.1086/692623 28641050

[B16] Dammhahn M. Kappeler P. (2008). Comparative feeding ecology of sympatric microcebus berthae and M-murinus. Int. J. Primatol. 29, 1567–1589. 10.1007/s10764-008-9312-318574599

[B17] Darwin C. R. (1874). The descent of man, and selection in relation to sex. London, UK: John Murray, Albermarle Street: tenth thousand 10.5962/bhl.title.54341

[B18] Dewar R. E. Richard A. F. (2007). Evolution in the hypervariable environment of Madagascar. Proc. Natl. Acad. Sci. U.S.A. 104, 13723–13727. 10.1073/pnas.0704346104 17698810 PMC1947998

[B19] Dewsbury D. A. (1982). Ejaculate cost and male choice. Am. Nat. 119, 601–610. 10.1086/283938

[B20] Divakaruni A. S. Rogers G. W. Murphy A. N. (2014). Measuring mitochondrial function in permeabilized cells using the seahorse XF analyzer or a clark-type oxygen electrode. Curr. Protoc. Toxicol. 60, 25.2.1–16. 10.1002/0471140856.tx2502s60 24865646

[B21] Emlen S. T. Oring L. W. (1977). Ecology, sexual selection, and the evolution of mating systems. Science 197, 215–223. 10.1126/science.327542 327542

[B22] Fournier F. Thomas D. W. Garland T. (1999). A test of two hypotheses explaining the seasonality of reproduction in temperate mammals. Funct. Ecol. 13, 523–529. 10.1046/j.1365-2435.1999.00342.x

[B23] Geiser F. Currie S. E. O'shea K. A. Hiebert S. M. (2014). Torpor and hypothermia: reversed hysteresis of metabolic rate and body temperature. Am. J. Physiol. Regul. Integr. Comp. Physiol. 307, R1324–R1329. 10.1152/ajpregu.00214.2014 25253085

[B24] Genin F. Perret M. (2003). Daily hypothermia in captive grey mouse lemurs (*Microcebus murinus*): effects of photoperiod and food restriction. Comp. Biochem. Physiol. B Biochem. Mol. Biol. 136, 71–81. 10.1016/S1096-4959(03)00172-6 12941640

[B25] Giroud S. Blanc S. Aujard F. Bertrand F. Gilbert C. Perret M. (2008). Chronic food shortage and seasonal modulations of daily torpor and locomotor activity in the grey mouse lemur (*Microcebus murinus*). Am. J. Physiol. Regul. Integr. Comp. Physiol. 294, R1958–1967. 10.1152/ajpregu.00794.2007 18434438

[B26] Grayson K. L. Mitchell N. J. Monks J. M. Keall S. N. Wilson J. N. Nelson N. J. (2014). Sex ratio bias and extinction risk in an isolated population of Tuatara (*Sphenodon punctatus*). PLoS One 9:e94214. 10.1371/journal.pone.0094214 24714691 PMC3979778

[B27] Guevara R. Santandreu F. M. Valle A. Gianotti M. Oliver J. Roca P. (2009). Sex-dependent differences in aged rat brain mitochondrial function and oxidative stress. Free Radic. Biol. Med. 46, 169–175. 10.1016/j.freeradbiomed.2008.09.035 18992805

[B28] Harcourt A. H. Harvey P. H. Larson S. G. Short R. V. (1981). Testis weight, body weight and breeding system in primates. Nature 293, 55–57. 10.1038/293055a0 7266658

[B29] Harman D. (1956). Aging: a theory based on free radical and radiation chemistry. J. Gerontol. 11, 298–300. 10.1093/geronj/11.3.298 13332224

[B30] Hohenbrink S. Koberstein-Schwarz M. Zimmermann E. Radespiel U. (2015). *Shades of gray mouse* lemurs: Ontogeny of female dominance and dominance-related behaviors in a nocturnal primate. Am. J. Primatol. 77, 1158–1169. 10.1002/ajp.22452 26212788

[B31] Humphries M. M. Kramer D. L. Thomas D. W. (2003). The role of energy availability in Mammalian hibernation: an experimental test in free-ranging eastern chipmunks. Physiol. Biochem. Zool. 76, 180–186. 10.1086/367949 12794671

[B32] Jonasson K. A. Willis C. K. (2011). Changes in body condition of hibernating bats support the thrifty female hypothesis and predict consequences for populations with white-nose syndrome. PLoS One 6:e21061. 10.1371/journal.pone.0021061 21731647 PMC3120823

[B33] Josse J. Husson F. (2016). missMDA: a package for handling missing values in multivariate data analysis. J. Stat. Softw. 70 10.18637/jss.v070.i01

[B34] Key C. Ross C. (1999). Sex differences in energy expenditure in non-human primates. Proc. R. Soc. Lond. B Biol. Sci. 266, 2479–2485. 10.1098/rspb.1999.0949 10693818 PMC1690481

[B35] Killick R. Eckley I. A. (2014). changepoint: an R package for changepoint analysis. J. Stat. Softw. 58, 1–19. 10.18637/jss.v058.i03

[B36] Kraus C. Eberle M. Kappeler P. M. (2008). The costs of risky male behaviour: sex differences in seasonal survival in a small sexually monomorphic primate. Proc. R. Soc. Lond. B Biol. Sci. 275, 1635–1644. 10.1098/rspb.2008.0200 18426751 PMC2602817

[B37] Kregel K. C. Zhang H. J. (2007). An integrated view of oxidative stress in aging: basic mechanisms, functional effects, and pathological considerations. Am. J. Physiol. Regul. Integr. Comp. Physiol. 292, R18–R36. 10.1152/ajpregu.00327.2006 16917020

[B38] Landes J. Henry P. Y. Hardy I. Perret M. Pavard S. (2019). Female reproduction bears no survival cost in captivity for gray mouse lemurs. Ecol. Evol. 9, 6189–6198. 10.1002/ece3.5124 31236213 PMC6580269

[B39] Le Galliard J. F. Fitze P. S. Ferriere R. Clobert J. (2005). Sex ratio bias, male aggression, and population collapse in lizards. Proc. Natl. Acad. Sci. U.S.A. 102, 18231–18236. 10.1073/pnas.0505172102 16322105 PMC1312374

[B40] Le S. Josse J. Husson F. (2008). FactoMineR: an R package for multivariate analysis. J. Stat. Softw. 25, 1–18. 10.18637/jss.v025.i01

[B41] Lennox A. R. Goodship A. E. (2008). Polar bears (*Ursus maritimus*), the most evolutionary advanced hibernators, avoid significant bone loss during hibernation. Comp. Biochem. Physiol. Part A Mol. Integr. Physiol. 149, 203–208. 10.1016/j.cbpa.2007.11.012 18249018

[B42] Levay E. A. Tammer A. H. Penman J. Kent S. Paolini A. G. (2010). Calorie restriction at increasing levels leads to augmented concentrations of corticosterone and decreasing concentrations of testosterone in rats. Nutr. Res. 30, 366–373. 10.1016/j.nutres.2010.05.001 20579529

[B43] Lighton J. R. B. (2018). Measuring Metabolic Rates: A Manual for Scientists, 2nd Ed. New York, NY: Oxford University Press 10.1093/oso/9780198830399.001.0001

[B44] Loft S. Fischernielsen A. Jeding I. B. Vistisen K. Poulsen H. E. (1993). 8-Hydroxydeoxyguanosine as a urinary biomarker of oxidative DNA-damage. J. Toxicol. Environ. Health Part A 40, 391–404. 10.1080/15287399309531806 8230310

[B45] Lusk G. (1924). Animal calorimetry twenty-fourth paper. analysis of the oxidation of mixtures of carbohydrate and fat. J. Biol. Chem. 59, 41–42.

[B46] Marin D. P. Bolin A. P. Dos Santos Rde C. Curi R. Otton R. (2010). Testosterone suppresses oxidative stress in human neutrophils. Cell Biochem. Funct. 28, 394–402. 10.1002/cbf.1669 20589735

[B47] Masoro E. J. (2005). Overview of caloric restriction and ageing. Mech. Ageing Dev. 126, 913–922. 10.1016/j.mad.2005.03.012 15885745

[B48] Massafra C. De Felice C. Gioia D. Buonocore G. (1998). Variations in erythrocyte antioxidant glutathione peroxidase activity during the menstrual cycle. Clin. Endocrinol. 49, 63–67. 10.1046/j.1365-2265.1998.00441.x 9797848

[B49] McAllan B. M. Geiser F. (2014). Torpor during reproduction in mammals and birds: dealing with an energetic conundrum. Integr. Comp. Biol. 54, 516–532. 10.1093/icb/icu093 24973362

[B50] Miller M. W. Hobbs N. T. Sousa M. C. (1991). Detecting stress responses in rocky-mountain bighorn sheep (*Ovis-Canadensis*-*Canadensis*) - reliability of cortisol concentrations in urine and feces. Can. J. Zool. 69, 15–24. 10.1139/z91-003

[B51] Muehlenbein M. P. Bribiescas R. G. (2005). Testosterone-mediated immune functions and male life histories. Am. J. Hum. Biol. 17, 527–558. 10.1002/ajhb.20419 16136532

[B52] Nakamura Y. Walker B. R. Ikuta T. (2016). Systematic review and meta-analysis reveals acutely elevated plasma cortisol following fasting but not less severe calorie restriction. Stress 19, 151–157. 10.3109/10253890.2015.1121984 26586092

[B53] Pamplona R. Costantini D. (2011). Molecular and structural antioxidant defenses against oxidative stress in animals. Am. J. Physiol. Regul. Integr. Comp. Physiol. 301, R843–863. 10.1152/ajpregu.00034.2011 21775650

[B54] Patel R. Williams-Dautovich J. Cummins C. L. (2014). Minireview: new molecular mediators of glucocorticoid receptor activity in metabolic tissues. Mol. Endocrinol. 28, 999–1011. 10.1210/me.2014-1062 24766141 PMC5414825

[B55] Perret M. Aujard F. (2001). Regulation by photoperiod of seasonal changes in body mass and reproductive function in gray mouse lemurs (*Microcebus murinus*): differential responses by sex. Int. J. Primatol. 22, 5–24. 10.1023/A:1026457813626

[B56] Perret M. Aujard F. Vannier G. (1998). Influence of daylength on metabolic rate and daily water loss in the male prosimian primate Microcebus murinus. Comp. Biochem. Physiol., Part A Mol. Integr. Physiol. 119, 981–989. 9773491 10.1016/s1095-6433(98)00015-4

[B57] Pifferi F. Terrien J. Marchal J. Dal-Pan A. Djelti F. Hardy I.. (2018). Caloric restriction increases lifespan but affects brain integrity in grey mouse lemur primates. Commun. Biol. 1:30. 10.1038/s42003-018-0024-8 30271916 PMC6123706

[B58] Qiu X. Brown K. Hirschey M. D. Verdin E. Chen D. (2010). Calorie restriction reduces oxidative stress by SIRT3-mediated SOD2 activation. Cell Metab. 12, 662–667. 10.1016/j.cmet.2010.11.015 21109198

[B59] R Core Team (2016). “R: A language and environment for statistical computing”. (Vienna, Austria: R Foundation for Statistical Computing).

[B60] Radespiel U. (2006). Ecological diversity and seasonal adaptations of mouse lemurs (microcebus spp.), in Lemurs, eds. Gould E. Sauther M.L. (Boston, MA: Springer), 211–234.

[B61] Radespiel U. Cepok S. Zietemann V. Zimmermann E. (1998). Sex-specific usage patterns of sleeping sites in grey mouse lemurs (Microcebus murinus) in northwestern Madagascar. Am. J. Primatol. 46, 77–84. 10.1002/(SICI)1098-2345(1998)46:1&lt;77::AID-AJP6&gt;3.0.CO;2-S 9730214

[B62] Radespiel U. Reimann W. Rahelinirina M. Zimmermann E. (2006). Feeding ecology of sympatric mouse lemur species in northwestern Madagascar. Int. J. Primatol. 27, 311–321. 10.1007/s10764-005-9005-0

[B63] Radespiel U. Sarikaya Z. Zimmermann E. Bruford M. (2001). Sociogenetic structure in a free- living nocturnal primate population: sex-specific differences in the grey mouse lemur (Microcebus murinus). Behav. Ecol. Sociobiol. 50, 493–502. 10.1007/s002650100402

[B64] Rodriguez-Cuenca S. Pujol E. Justo R. Frontera M. Oliver J. Gianotti M.. (2002). Sex-dependent thermogenesis, differences in mitochondrial morphology and function, and adrenergic response in brown adipose tissue. J. Biol. Chem. 277, 42958–42963. 10.1074/jbc.M207229200 12215449

[B65] Ruiz E. Jackson S. Cimentada J. (2019). corrr: Correlations in R. Avaliable onine at: https://CRAN.R-project.org/package=corrr

[B66] Schmid J. Kappeler P. M. (1998). Fluctuating sexual dimorphism and differential hibernation by sex in a primate, the gray mouse lemur (*Microcebus murinus*). Behav. Ecol. Sociobiol. 43, 125–132.

[B67] Shine R. (1989). Ecological causes for the evolution of sexual dimorphism: a review of the evidence. Q. Rev. Biol. 64, 419–461. 10.1086/416458 2697022

[B68] Slominski A. T. Zmijewski M. A. Skobowiat C. Zbytek B. Slominski M. Steketee J. D. (2013). Sensing the environment: regulation of local and global homeostasis by the skin neuroendocrine system. Adv. Anat. Embryol. Cell Biol. 98. 10.1007/978-3-642-19683-6 22894052 PMC3422784

[B69] Sohal R. S. Weindruch R. (1996). Oxidative stress, caloric restriction, and aging. Science 273, 59–63. 10.1126/science.273.5271.59 8658196 PMC2987625

[B70] Sugioka K. Shimosegawa Y. Nakano M. (1987). Estrogens as natural antioxidants of membrane phospholipid peroxidation. FEBS Lett. 210, 37–39. 10.1016/0014-5793(87)81293-0 3803578

[B71] Terrien J. Gaudubois M. Champeval D. Zaninotto V. Roger L. Riou J. F.. (2017). *Metabolic and genomic adaptations to winter fattening in a primate* species, the grey mouse lemur (Microcebus murinus). Int. J. Obes. 42, 221–230. 10.1038/ijo.2017.195 28925409

[B72] Thomsen R. Soltis J. Matsubara M. Matsubayashi K. Onuma M. Takenaka O. (2006). How costly are ejaculates for Japanese macaques? Primates 47, 272–274. 10.1007/s10329-005-0171-7 16467956

[B73] Trivers R. L. (1972). Parental investment and sexual selection, in Sexual Selection and the Descent of Man, 1871-1971, ed Campbell B. (Chicago, IL: Aldine), 136–179.

[B74] Valle A. Garcia-Palmer F. J. Oliver J. Roca P. (2007). Sex differences in brown adipose tissue thermogenic features during caloric restriction. Cell. Physiol. Biochem. 19, 195–204. 10.1159/000099207 17310113

[B75] Villain N. Picq J. L. Aujard F. Pifferi F. (2016). Body mass loss correlates with cognitive performance in primates under acute caloric restriction conditions. Behav. Brain Res. 305, 157–163. 10.1016/j.bbr.2016.02.037 26952885

[B76] Vuarin P. Dammhahn M. Kappeler P. M. Henry P. Y. (2015). When to initiate torpor use? food availability times the transition to winter phenotype in a tropical heterotherm. Oecologia. 179, 43–53. 10.1007/s00442-015-3328-0 25953115

[B77] Wedell N. Gage M. J. G. Parker G. A. (2002). Sperm competition, male prudence and sperm-limited females. Trends Ecol. Evol. 17, 313–320. 10.1016/S0169-5347(02)02533-818588428

[B78] Wei Y. Zhang J. Xu S. Peng X. Yan X. Li X.. (2018). Controllable oxidative stress and tissue specificity in major tissues during the torpor-arousal cycle in hibernating Daurian ground squirrels. Open Biol. 8:180068. 10.1098/rsob.180068 30305429 PMC6223210

[B79] Wells J. C. (2000). Natural selection and sex differences in morbidity and mortality in early life. J. Theor. Biol. 202, 65–76. 10.1006/jtbi.1999.1044 10623500

[B80] Willis C. K. R. (2017). Trade-offs influencing the physiological ecology of hibernation in temperate-zone bats. Integr. Comp. Biol. 57, 1214–1224. 10.1093/icb/icx087 28985332

[B81] Wrogemann D. Radespiel U. Zimmermann E. (2001). Comparison of reproductive characteristics and changes in body weight between captive populations of rufous and gray mouse lemurs. Int. J. Primatol. 22, 91–108. 10.1023/A:1026418132281

[B82] Zeveloff S. I. Boyce M. S. (1980). Parental investment and mating systems in mammals. Evolution 34, 973–982. 10.1111/j.1558-5646.1980.tb04035.x 28581141

[B83] Zhao X. Psarianos P. Ghoraie L. S. Yip K. Goldstein D. Gilbert R. (2019). Metabolic regulation of dermal fibroblasts contributes to skin extracellular matrix homeostasis and fibrosis. Nat. Metab. 1, 147–157. 10.1038/s42255-018-0008-5 32694814

