# Supplementary figures and images for: Sex-Specific Response to Caloric Restriction After Reproductive Investment in Microcebus murinus: An Integrative Approach
Source: Front Physiol. 2020 Jun 16;11:506. doi: 10.3389/fphys.2020.00506 (PMC7308708; doi:10.3389/fphys.2020.00506)

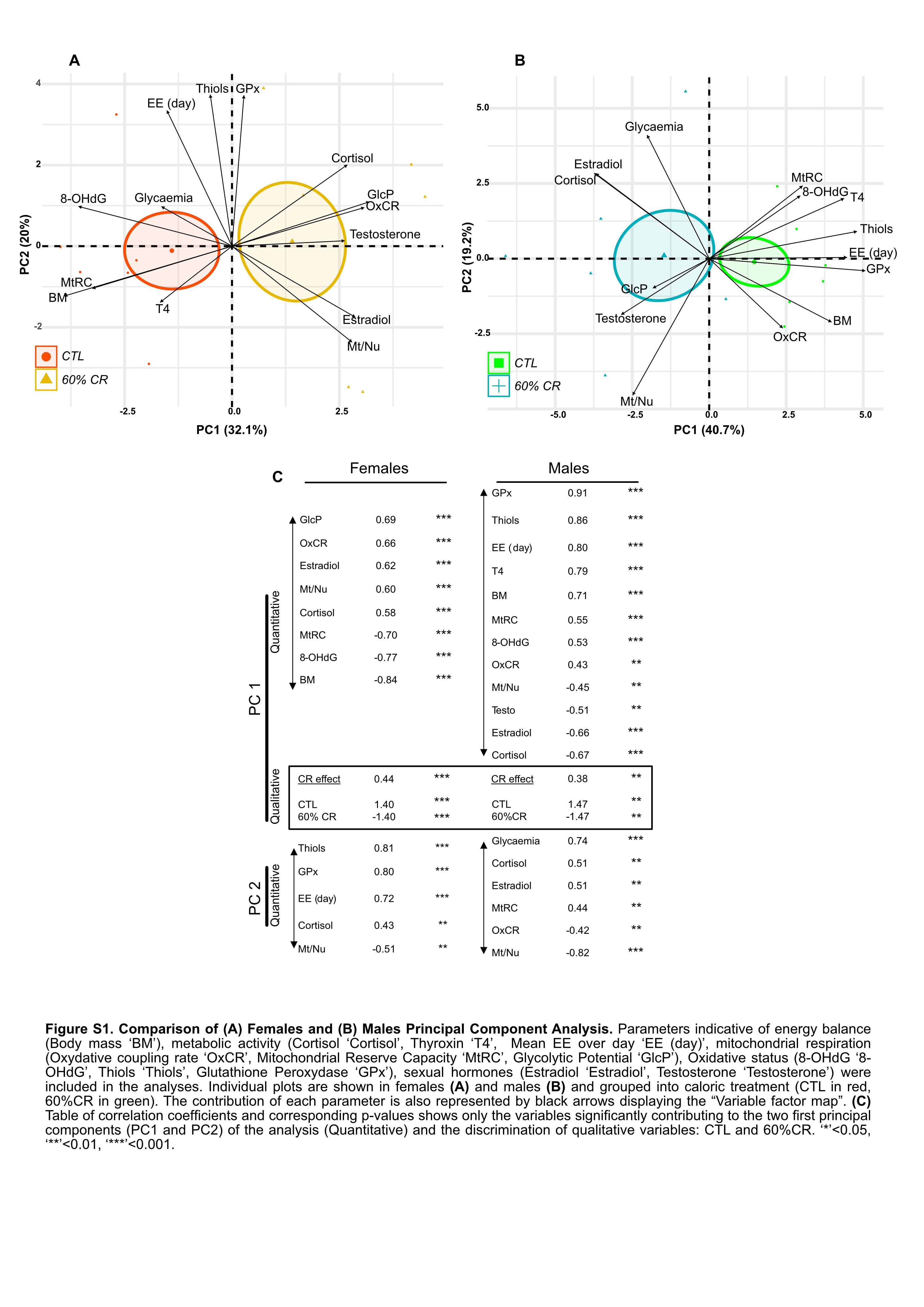

Supplement: Figure S1 — Comparison of (A) Females and (B) Males Principal Component Analysis. Parameters indicative of energy balance (Body mass “BM”), metabolic activity (Cortisol “Cortisol,” Thyroxin “T4,” Mean EE over day “EE (day),” mitochondrial respiration (Oxydative coupling rate “OxCR,” Mitochondrial Reserve Capacity “MtRC,” Glycolytic Potential “GlcP”), Oxidative status (8-OHdG “8-OHdG,” Thiols “Thiols,” Glutathione Peroxydase “GPx”), sexual hormones (Estradiol “Estradiol,” Testosterone “Testosterone”) were included in the analyses. Individual plots are shown in females (A) and males (B) and grouped into caloric treatment (CTL in red, 60%CR in green). The contribution of each parameter is also represented by black arrows displaying the “Variable factor map”. (C) Table of correlation coefficients and corresponding p-values shows only the variables significantly contributing to the two first principal components (PC1 and PC2) of the analysis (Quantitative) and the discrimination of qualitative variables: CTL and 60%CR. * <0.05, ** <0.01, *** <0.001. [file Image_1.jpeg]

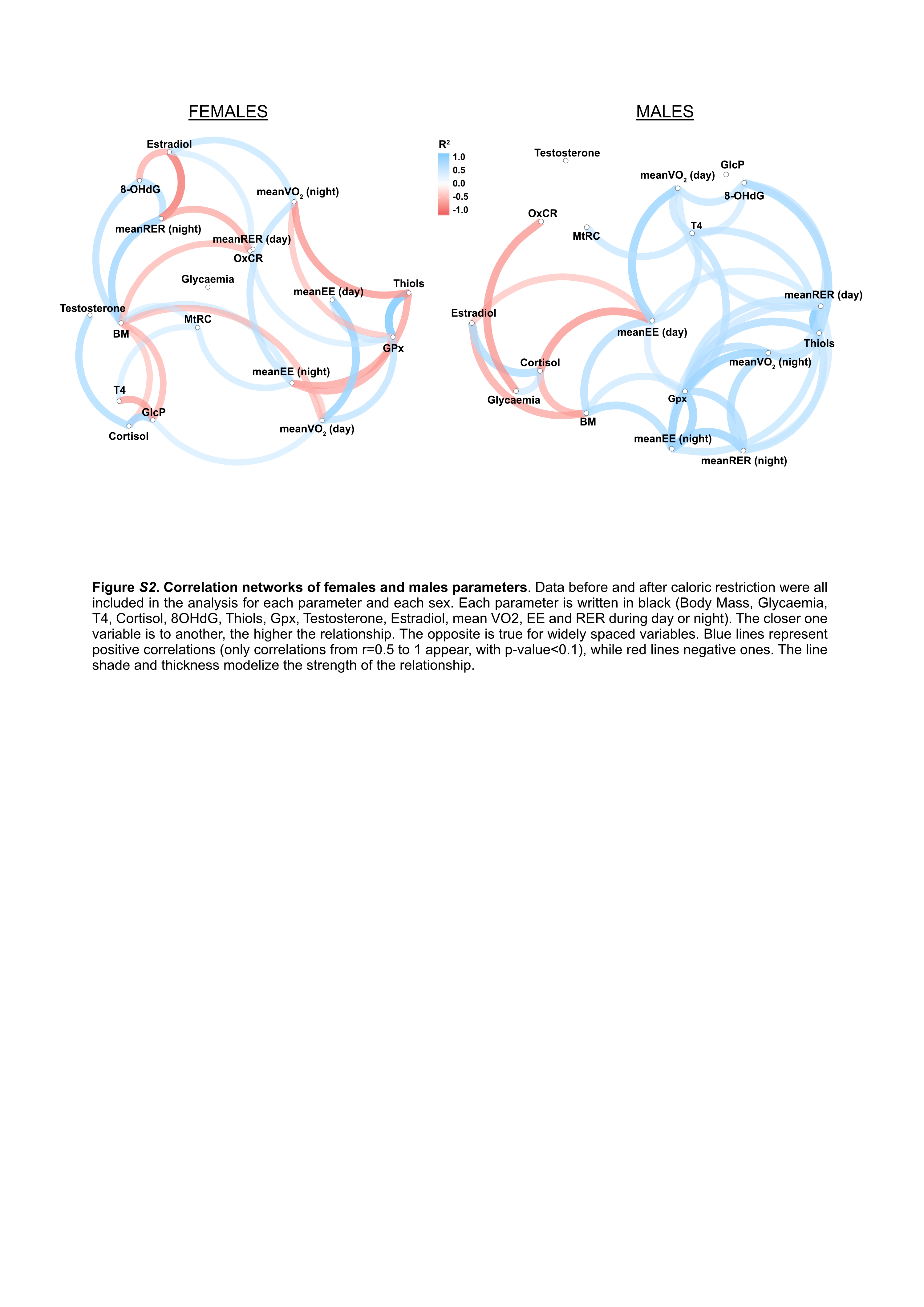

Supplement: Figure S2 — Correlation networks of females and males parameters. Data before and after caloric restriction were all included in the analysis for each parameter and each sex. Each parameter is written in black (Body Mass, Glycaemia, T4, Cortisol, 8OHdG, Thiols, Gpx, Testosterone, Estradiol, mean VO2, EE and RER during day or night). The closer one variable is to another, the higher the relationship. The opposite is true for widely spaced variables. Blue lines represent positive correlations (only correlations from r = 0.5 to 1 appear, with p < 0.1), while red lines negative ones. The line shade and thickness modelize the strength of the relationship. [file Image_2.jpeg]
